# Supplementary material for: Preservation versus resection of Denonvilliers’ fascia in total mesorectal excision for male rectal cancer: follow-up analysis of the randomized PUF-01 trial
Source: Nat Commun. 2023 Oct 20;14:6667. doi: 10.1038/s41467-023-42367-3 (PMC10589235; doi:10.1038/s41467-023-42367-3)
Supplement: Supplementary file 1 — Supplementary Information [file 41467_2023_42367_MOESM1_ESM.pdf]

Supplementary Information

Supplementary Table 1. Comparison of Urinary Function in the Per-protocol Population

Parameter	Exp-Group		Con-Group		*P*-value
	n	n (%) or Mean (SD)	n	n (%) or Mean (SD)	
RUV（mL）*					
Preoperative	92	12.41(13.50)	86	16.75(18.79)	0.081
POW2	80	34.80(54.02)	70	75.11(84.07)	0.001
POM3	54	20.99(23.10)	55	37.17(27.40)	0.001
POM6	51	12.33(10.85)	40	37.05(34.78)	< 0.001
Urinary dysfunction rate†					
POW2	80	5(6.3%)	67	17(25.7%)	0.001
POM3	65	3(4.6%)	65	6(9.2%)	0.492
POM6	62	(0%)	52	3(5.8%)	0.092
Max-UFR*					
Preoperative	70	18.05(8.47)	68	17.79(7.66)	0.849
POW2	64	15.93(7.86)	62	12.26(6.92)	0.006
POM3	44	17.54(6.92)	48	14.96(7.21)	0.084
POM6	43	18.30(7.32)	35	14.17(7.03)	0.014
IPSS*					
Preoperative	100	3.49(2.75)	93	4.34(4.29)	0.104
POW2	92	6.52(5.71)	87	8.55(5.78)	0.019
POM3	77	5.34(4.76)	73	7.03(5.53)	0.046
POM6	67	5.18(4.37)	51	6.43(5.85)	0.185

Abbreviations: SD, standard deviation; RUV, residual urine volume; MFR, maximal flow rate; IPSS, International Prostate Symptom Score; POM, Postoperative Month; POW, Postoperative week.

* Quantitative data are calculated by two-sided t-test.

† Qualitative data using Pearson's or Cochran-Mantel-Haenszel two-sided $\chi 2$-test.

Supplementary Table 2. Comparison of Erectile and Ejaculation Functions in the Per-protocol Population

Parameter	Exp-Group		Con-Group		*P*-value
	n	n (%) or Mean (SD)	n	n (%) or Mean (SD)	
IIEF5*					
Preoperative	99	17.36(6.57)	90	16.96(7.31)	0.687
POM1	90	15.78(6.11)	84	12.85(12.69)	0.051
POM3	73	15.25(6.64)	71	12.41(6.40)	0.010
POM6	63	15.46(6.49)	52	11.79(6.04)	0.002
POM12	47	16.19(5.96)	41	12.88(6.44)	0.014
POM12 Erectile dysfunction rate (≤11score) †	47	6(12.8%)	41	16(39.0%)	0.005
Ejaculation dysfunction rate †					
POM1	78	11(14.1%)	69	21(30.4%)	0.017
POM3	70	10(14.3%)	65	19(29.2%)	0.035
POM6	54	6(11.1%)	44	14(31.8%)	0.011
POM12	47	4(8.5%)	37	11(29.7%)	0.012

Abbreviations: IIEF-5, the International Index of Erectile Function 5; POM, Postoperative Months

* Quantitative data are calculated by two-sided t-test.

† Qualitative data using Pearson’s or Cochran-Mantel-Haenszel two-sided $\chi 2$-test.

Supplementary Table 3. Postoperative Recovery Data of Patients in the Modified Intention-to-Treat Population

Postoperative Recovery Data	Exp-group (n=122)	Con-group (n=120)	*P* value
First flatus (h)	35.5±20.8	37.7±25.2	0.468
First liquid diet (h)	44.7±31.0	43.6±31.2	0.772
First semi-liquid diet (h)	68.8±40.2	64.0±39.1	0.352
Remove of drainage tube (h)	59.2±46.7	59.0±62.7	0.979
Remove of catheter (h)	38.3±38.8	47.5±34.8	0.053

The comparison of postoperative recovery data is calculated by two-sided t-test.

Supplementary Table 4. Surgery-related Data of Patients in the Modified Intention-to-Treat Population

Perioperative data	Exp-group (n=122)	Con-group (n=120)	*P* value
Surgical time (min) *	203.6±55.2	200.8±59.4	0.696
Intraoperative bleeding (ml) *	49.8±53.2	50.3±44.8	0.938
Surgical procedure†			0.271
LAR	112 (91.8%)	105 (87.5%)	
APR	10 (8.2%)	15 (12.5%)	
Conversion to open surgery	0	0	
Defunctioning stoma for LAR†	9 (8.0%)	11 (10.5%)	0.535
30-day mortality	0	0	
Total incidence of postoperative complications†	22 (18.0%)	21 (17.5%)	0.914
Intraoperative complication			
Hypercapnia	1 (0.8%)	1 (0.8%)	
Postoperative complication			
Anastomotic stenosis	2 (1.8%)	2 (1.9%)	
Anastomotic leakage	9 (8.0%)	8 (7.6%)	
Anastomotic bleeding	2 (1.8%)	1 (1.0%)	
Stoma bleeding/ischemia/stenosis	2 (10.5%)	3 (11.5%)	
Digestive tract infection	0 (0%)	1 (0.8%)	
Intestinal obstruction	2 (1.6%)	1 (0.8%)	
Catheter-related Septic shock	1 (0.8%)	0 (0%)	
Urinary infection	1 (0.8%)	1 (0.8%)	
Incisional infection	2 (1.6%)	2 (1.7%)	
Incisional hernia	0 (0%)	1 (0.8%)	

LAR: low anterior resection; APR: abdominal perineal resection.

* Quantitative data are calculated by two-sided t-test.

† Qualitative data using Pearson's two-sided χ2-test.

Supplementary Table 5. Univariate Analysis of Overall Survival at 3 Years' Follow-up

Variable	Patients No.	Exp-group, 3-y OS (95% CI), %	Patients No.	Con-group, 3-y OS (95% CI), %	Hazard ratio*	Log-rank *P* value
Total	122	94.1(90.0-98.4)	120	89.7(84.3-95.4)	0.56(0.22-1.42)	0.219
T stage						
T_{1-2}	38	97.3(92.2-100.0)	43	93.0(85.7-100.0)	0.37(0.04-3.57)	0.391
T_{3-4}	84	92.7(87.2-98.5)	77	87.8(80.6-95.6)	0.59(0.21-1.66)	0.320
N stage						
N_0	78	96.0(91.7-100.0)	68	92.6(86.5-99.1)	0.53(0.13-2.20)	0.379
N_1	30	89.9(79.6-100.0)	30	82.6(69.8-97.7)	0.54(0.13-2.28)	0.404
N_2	14	92.9(80.3-100.0)	22	90.5(78.8-100.0)	0.76(0.07-8.43)	0.826
TNM stage						
I	33	96.9(91.0-100.0)	35	94.3(86.9-100.0)	0.53(0.05-5.80)	0.599
II	45	95.4(89.4-100.0)	33	90.7(81.2-100.0)	0.51(0.08-3.04)	0.457
III	44	90.7(82.5-99.8)	52	85.8(76.7-96.1)	0.62(0.18-2.13)	0.451
Tumor location						
Anterior	28	NA	33	93.9(86.1-100.0)	NA	NA
Non-anterior	94	92.4(87.1-98.0)	87	88.0(81.4-95.3)	0.63(0.24-1.65)	0.346

* Reference, Con-group.

Supplementary Table 6. Eligibility Criteria for Enrolling Patients

**Criteria**	**Items**
**Inclusion criteria**	Male, 20 < age (years) < 71, informed consent;
	Pathological diagnosis of rectal adenocarcinoma;
	Tumors from anal edge 6 ~ 12 cm (measured by rigid proctoscope);
	Preoperative staging T_{1-4} (T_{1-2} for anterior rectal wall) $N_{0-2}M_0$ rectal cancer (AJCC- 7th);
	R_0 TME surgical results is expected;
	Preoperative ECOG physical status score 0/1;
	Preoperative ASA grade I ~ III;
	Normal urinary function (Bladder residual urine **<** 100ml), normal erection function (IIEF-5>21) and ejaculation function grading as I level.
**Exclusion criteria**	Complicated with acute ileus, perforation or hemorrhage;
	Tumors with extensive invasion of surrounding tissues, TME not applicable;
	With neoadjuvant radiotherapy;
	Imaging examination in regional integration intumescent lymph nodes (maximum diameter 3 cm or higher);
	With other malignant diseases or with other malignant disease within 5 years;
	With other diseases need surgery;
	A history of abdominal and pelvic major operation;
	People with severe mental illness, or cannot be evaluated due to cultural or psychological factors;
	No sexual life;
	Critical organ dysfunction, unbearable surgery;
	Unstable angina, myocardial infarction, cerebral infarction or hemorrhage within 6 months;
	Systemic corticosteroids or immunosuppressive medication history within 1 month;
	Pre-existent true incontinence or severe stress urinary incontinence.
**Exit criteria**	Confirmed as M_1 during or after operation;
	Conversion to abdominoperineal resection (APR)
	Postoperatively confirmed as invading rectal intrinsic fascia, or T3 for anterior rectal wall;

	Intraoperative confirmed regional lymph node fusion conglobation cannot ensure R_0 resection;
	Infiltrating major blood vessel and unresectable;
	Intraoperative finding other diseases need simultaneous surgery;
	Preoperative emergent severe complications, cannot carry out the study treatment;
	Emergency surgery is needed;
	Into this study, at any stage of the initiative exit or discontinue treatment;
	Prove to implement the healer violates this research plan.

Supplementary Note

Trial Protocol

Prospective Multicenter Randomized controlled Clinical Trial for Comparison of the Preservative Effect on Postoperative Urogenital Function between Denonvilliers' Fascia Preservation and Resection during Laparoscopic TME for Male Rectal Cancer Patients

(PUF 01, L-DVF-P vs L-DVF-R)

Clinical Study Scheme

Confidentiality Statement: The information contained in this study is only available for review by the researcher, ethics committee and relevant institutions. Without the approval of the principal investigator (PI), it is strictly forbidden to inform any third party that is not involved in the project.

SUMMARY OF RESEARCH PROGRAMS

**Project Title**：	Prospective Multicenter Randomized controlled Clinical Trial for Comparison of the Preservative Effect on Postoperative Urogenital Function between Denonvilliers' Fascia Preservation and Resection during Laparoscopic TME for Male Rectal Cancer Patients
**Version**	Version 1.02
**Research Centers**：	1. The Third Affiliated Hospital, Sun Yat-sen University; 2.Anyang cancer Hospital; 3. The Second Affiliated Hospital, Sun Yat-sen University; 4.The Sixth Affiliated Hospital, Sun Yat-sen University; 5. Affiliated Hospital of GuangDong Medical University; 6.Shantou Central Hospital; 7. The Second Affiliated Hospital of Guangzhou Medical University; 8. Affiliated Hospital, Henan University of Science and Technology; 9. Shunde Hospital, Southern Medical University; 10. The First Affiliated Hospital of Xiamen University; 11. Nanfang Hospital, Southern Medical University; 12. School of Public Health, Sun Yat-sen University
**Number of**	11

**Research Centers**:	
**Indication:**	Male patients with middle-low rectal cancer, preoperative staging T1-4 (T1-2 for anterior rectal wall) N0-2M0 (AJCC-7th)
**Purpose of Research :**	The protective effect of Denonvilliers' fascia (DVF) preservation during laparoscopic radical rectectomy (LR) on pelvic autonomic nerves (PAN) and the postoperative urination-sexual function remains controversial. We conducted a randomized controlled trial to compare the preservative effect of LR with DVF preservation (DVFP) and resection (DVFR) on urination-sexual function of male mid-low rectal cancer patients.
**Research Design:**	A prospective, multicenter, single-blind, randomized design was adopted with open, positive parallel control, and validation of superiority. The research met the ethical requirements.<br><br>Stratified blocked randomization was used, stratification factor was center and the block size was 4. The random

	allocation sequence was generated by a statistician who was independent of the research, using the SAS 9.3 software (SAS Institute, Cary, NC). Case enrollment is reviewed by the surgeon according to the inclusion and exclusion criteria. Subsequently, eligible patients were randomly assigned to the experimental (Exp-group) or control group (Con-group) by the surgeon using random envelopes, and then underwent L-TME with DVF preservation (L-DVF-P) or L-TME with DVF resection (L-DVF-R), respectively.<br><br>A single-blind design is adopted in this study. The participants and research assistants who participated in patient follow-up and functional evaluation were blinded, and the surgeons were informed of enrollment information before the operation.
**Grouping:**	Experimental group (Exp-Group): DVF preservation during laparoscopic total Mesorectal Excision (L-TME); control group (Con- Group): DVF resection during L-TME.
**Sample Size Determination**	In this study, the incidences of urinary dysfunction 2 weeks after operation and sexual dysfunction 12 months after operation were the main effective evaluation indicators. Our previous results showed that the incidence of urinary

	dysfunction was 24.39% at 2 weeks after DVFP-TME operation and that of sexual dysfunction was 9.76% 12 months after operation. The corresponding incidence for L-DVFR-TME, was 44.68% and 42.55%, respectively.<br><br>This study hypothesizes that the incidence of urinary dysfunction at 2 weeks and sexual dysfunction at 12 months after operation in the experimental group will be the same as that in the control group. The first two indicators were tested unilaterally, with αvalue of 5% and a value of 0.025 as a unilateral statistical significance level. A test efficiency of 90% was set, and a balanced design was adopted. Computational formula of unilateral tests:<br><br>$$n_1 = \frac{[Z_\alpha\sqrt{p(1-p)(1+c)/c} + Z_\beta\sqrt{p_1(1-p_1)+p_2(1-p_2)/c}]^2}{(p_1-p_2)^2}$$<br><br>The largest sample size was obtained by unilateral urinary dysfunction test, which required 110 samples for each group. Considering that the largest abscission rate in this clinical study was about 10%, the final sample size of each group was 121, and the total number of cases needed was 242. According to the established research plan, the preliminary functional evaluation results will be reported after

	enrollment is completed.
Inclusion criteria	● Male, 20 yrs < age < 71 yrs, informed consent.<br>● Pathological diagnosis of rectal adenocarcinoma.<br>● Tumors from anal edge 6 ~ 12 cm, (measured by rigid proctoscope).<br>● Preoperative staging T_{1-4} (T_{1-2} for anterior rectal wall) $N_{0-2}M_0$ rectal cancer (AJCC- 7th).<br>● R_0 TME surgical results are expected.<br>● Preoperative ECOG physical status score 0/1.<br>● Preoperative ASA grade I ~ III.<br>● Normal urinary function (bladder residual urine＜100 ml), normal erection function (IIEF-5>21) and ejaculation function grading as I level.
**Exclusion criteria**	● Complication with acute ileus, perforation or hemorrhage<br>● Tumors with extensive invasion of surrounding tissues, TME not applicable.<br>● With neoadjuvant radiotherapy;<br>● Imaging examination in regional integration intumescent lymph nodes (maximum diameter 3 cm or larger).<br>● With other malignant diseases or with other malignant disease within 5 years.<br>● With other diseases needing surgery.<br>● A history of abdominal or pelvic major operation.<br>● People with severe mental illness, or that cannot be evaluated due to cultural or psychological factors.<br>● No sexual life.<br>● Critical organ dysfunction, unbearable surgery.<br>● Unstable angina, myocardial infarction, cerebral infarction or hemorrhage within 6 months.<br>● Systemic corticosteroids or immunosuppressive medication history within 1 month.

	• Pre-existent true incontinence or severe stress urinary incontinence.
**Exit criteria**	• Confirmed as M_1 during or after operation.<br>• Conversion to abdominoperineal resection (APR).<br>• Postoperatively confirmed as invading rectal intrinsic fascia, or T3 for anterior rectal wall.<br>• Intraoperative confirmed regional lymph node fusion conglobation cannot ensure R_0 resection.<br>• Infiltrating major blood vessel and unresectable.<br>• Intraoperative finding of other diseases needing simultaneous surgery.<br>• Preoperative emergent severe complications cannot undergo the study treatment.<br>• Emergency surgery is needed.<br>• Into this study, at any stage of the initiative exit or discontinue treatment.<br>• Prove to implement the treatment violates this research plan.
**Intervention**	Low rectal anterior resection was performed according to the " Criteria for diagnosis and treatment of colorectal cancer (2010)" and " Surgical clinical pathway for Low Anterior Rectal Cancer" of the Ministry of Health in China, and NCCN Guidelines for the Treatment of Colorectal Cancer (2014 edition). Meanwhile, PANP was performed according to the modified Sugihara-PANP classification<br>• Experimental group: DVF preservation during laparoscopic radical rectectomy (LR);<br>• Control group: DVF resection during laparoscopic radical rectectomy (LR).
**Study**	• **Primary study endpoint**

**Endpoint**	Incidence of urinary dysfunction 2 weeks after operation and sexual dysfunction (erectile and ejaculation dysfunction) 12 months after operation.<br>● **Secondary study endpoint**<br>➢ 1,3-year overall survival rate<br>➢ 1,3-year disease free survival rate<br>➢ 1,3-year recurrence and recurrence pattern<br>➢ Morbidity and mortality (30 days postoperative) rates<br>➢ Postoperative recovery course

1. BACKGROUND

Colorectal cancer (CRC) is one of the most common malignant tumors of the digestive tract, and seriously threatens human health. The incidence of colorectal cancer in China is increasing every year. Rectal cancer affects half of the country, and the majority of the cases are of middle and low rectal cancer. Currently, the treatment of middle and low rectal cancer is still a comprehensive treatment based on surgical resection. Before 1980s, the local recurrence rate of rectal cancer was very high after surgery. Then, the principle of total mesorectal excision (TME) was put forward and it became the golden standard of operation for middle and low rectal cancer. Thanks to this technique the local recurrence rate and long-term survival of rectal cancer achieved "revolutionary" progress.

However, due to pelvic autonomic nerve (PAN) injury, the incidence of urinary and sexual dysfunction after TME is as high as 7% - 70% and 40% - 100%, respectively. Both of these conditions seriously affect the quality of life of patients after TME [3-5]. Studies have shown that radical resection of rectal cancer with pelvic autonomic nerve protection (PANP) can reduce the incidence of urinary and sexual dysfunction to a certain extent, but it is still unsatisfactory.

At present, laparoscopic radical rectectomy has been authorized by the academic community, for its feasibility, safety and long-term efficacy. Laparoscopy enlarges the clear surgical field of vision and enables surgeons

to perform fine operations in a narrow pelvic cavity under direct vision. It also makes the choice of the surgical plane and the identification of the structure clearer, and makes the real PANP operation possible [10-14]. Since 1990s, the PI on this project has devoted himself to this research field [15-17], and has been carrying out laparoscopic radical rectectomies with PANP since the early 2000s. This approach has allowed the incidence of urinary dysfunction to be reduced to 18%, while that of erectile and ejaculatory dysfunction has been reduced to 14% and 16%, respectively [18].

Our systematic study of PAN branches and their route, by means of autopsy, showed that the abdominal aortic plexus passes through the anterior and bilateral sides of the abdominal aorta, and continues downward under the Toldt space into the superior hypogastric plexus (SHP). There is a potential gap between the two layers of the pelvic fascia visceral wall, which appears as a complete "bucket" around the mesorectal, in which the left and right hypogastric nerves (HN) from SHP descend. To both sides of the rectum, S2-4 sacral parasympathetic fibers are known to form the inferior hypogastric plexus (IHP), which is transferred to the front through the lateral rectal ligament and into the bladder and prostate [19,20] in the front of Denonvilliers' fascia (DVF). Based on the above research, we put forward the "three-gap theory", and clearly pointed out that the posterior mesorectal space and DVF space (anterior rectal space) belong to the first space, which is also the

anatomical basis of TME. This observation coincides with the latest studies of embryogenesis and histopathology [21, 22].

The choice of surgical plane during TME is the key factor to determine whether PAN is damaged or not. There is no controversy in the protection of the PAN branches of SHP, HN and lateral IHP. The controversy focuses on the pelvic plexus anterior rectum (i.e. the efferent branches of IHP). The protection of efferent branches of the IHP is associated with the selection of the surgical plane and the understanding of the anatomy and function of DVF. Lindsey et al. proposed three potential surgical planes for TME surgery: (1) mesangial plane, i.e. separation in the mesorectal membrane during surgery; (2) the mesorectal plane, i.e., behind DVF, operates along the rectal fascia propria; and (3) the outer mesorectal plane, i.e., completes TME in front of DVF. Obviously, the first surgical plane destroyed the integrity of the mesorectal, which was inconsistent with the TME principle. Therefore, the current debate on TME for rectal cancer is whether to choose DVF anterior or posterior (i.e. resection or preservation of DVF) for mesorectal excision. Heald, the “father” of TME, believed that DVF and proper rectal fascia were densely adhered and could not be separated. Therefore, complete removal of DVF was necessary for TME. In addition, he believes that TME with DVF preservation in patients with locally advanced tumors will lead to an increase in local recurrence rate [23].

However, an increasing number of studies, using autopsy and fresh corpse surgeries, contradict the classical TME principle. At the level of the male seminal vesicle gland, neurovascular bundles traversed in front of DVF, which were found by experimental models and 3D reconstruction techniques. The PAN branch is closely related to urogenital function [24, 25]. Rectal and mesorectal excision was performed in front of DVF. Damages to these nerves and vascular bundles (NVB) lead to urinary and sexual dysfunction. However, DVF and neurological function are currently interlinked. Research in the field of energy is limited to basic research of anatomical histoembryology and earlier clinical oncology studies. In clinical work, the difference between the effect of the preservation or removal of DVF on the protection of urinary and sexual function for specific rectal cancer patients undergoing TME is rarely reported.

In our previous work, we performed LR with DVF preservation for specific rectal cancer patients (T1-2N0M0) to explore the effect of this operation on male urinary-sexual function and radical cure of tumors. Preliminary results showed that, compared with the classic LR with DVF resection, patients who underwent LR with DVF preservation displayed better bladder contractility, IPSS, erectile and ejaculatory function scores. Results suggest that preservation of DVF can offer better protection of male urination and sexual function. At the same time, there was no statistical difference in the 3 year survival between the two groups, suggesting that preservation of DVF did not increase the incidence of local recurrence rate [26].

Based on the above results, a large sample, multi-center randomized controlled study was conducted. The main purpose of this study was to investigate the quality of life and long-term survival rate of patients with rectal cancer after DVF preservation through the quantitative evaluation of urodynamics, IPSS score and sexual function indicators, and long-term and meticulous follow-up work, and to further determine the rationality and feasibility of the procedure. If this project is successful, it will bring about a major breakthrough in radical surgery for rectal cancer, and improve the quality of life of tens of thousands of patients undergoing surgery for rectal cancer, which will surely bring huge social benefits.

Reference

1. Siegel RL, Miller KD, Jemal A. Cancer statistics, 2015. CA Cancer J Clin. 2015, 65(1):5-29.

2. Bonjer HJ, Deijen CL, Abis GA, Cuesta MA, van der Pas MH, de Lange-de Klerk ES, Lacy AM, Bemelman WA, Andersson J, Angenete E, Rosenberg J, Fuerst A, Haglind E; COLOR II Study Group. A randomized trial of laparoscopic versus open surgery for rectal cancer. N Engl J Med. 2015 Apr 2; 372(14):1324-32.

3. Khair MA, Rahman MS, Khanam F, Haque S, Hoque MM, Rahman MM, Paul BK, Kundu GK. Sexual and urinary outcome after surgical treatment for low rectal cancer. Mymensingh Med J. 2013, 22(3): 444-451.

4. Kasparek MS, Hassan I, Cima RR, Larson DR, Gullerud RE, Wolff BG. Long-term quality of life and sexual and urinary function after abdominoperineal resection for distal rectal cancer. Dis Colon Rectum. 2012, 55(2): 147-154.

5. Contin P, Kulu Y, Bruckner T, Sturm M, Welsch T, Müller-Stich BP, Huber J, Büchler MW, Ulrich A. Comparative analysis of late functional outcome following

preoperative radiation therapy or chemoradiotherapy and surgery or surgery alone in rectal cancer. Int J Colorectal Dis. 2014, 29(2):165-175.

6. D. Moszkowicz, B. Alsaid, T. Bessede, C. Penna, B. Nordlinger, G. Benoıˆt, F. Peschaud. Where does pelvic nerve injury occur during rectal surgery for cancer? Colorectal Disease, 2011,13: 1326-1334.

7. Kneist W, Kauff DW, Juhre V, Hoffmann KP, Lang H. Is intraoperative neuromonitoring associated with better functional outcome in patients undergoing open TME? Results of a case-control study. EJSO 2013, 39: 994-999.

8. Kauff DW, Koch KP, Somerlik KH, Hoffmann KP, Lang H, Kneist W. Evaluation of two-dimensional intraoperative neuromonitoring for predicting urinary and anorectal function after rectal cancer surgery. Int J Colorectal Dis 2013, 28:659–664.

9. Runkel N, Reiser H. Nerve-oriented mesorectal excision (NOME): autonomic nerves as landmarks for laparoscopic rectal resection. Int J Colorectal Dis 2013, 28:1367-1375.

10. McGlone ER, Khan O, Flashman K, Khan J, Parvaiz A. Urogenital function following laparoscopic and open rectal cancer resection: a comparative study. Surgical Endoscopy. 2012, 26(9): 2559-2565.

11. Kim JY, Kim NK, Lee KY, Hur H, Min BS, Kim JH. A comparative study of voiding and sexual function after total mesorectal excision with autonomic nerve preservation for rectal cancer: laparoscopic versus robotic surgery. Ann Surg Oncol. 2012, 19(8): 2485-2493.

12. Luca F, Valvo M, Ghezzi TL, Zuccaro M, Cenciarelli S, Trovato C, Sonzogni A, Biffi R. Impact of Robotic Surgery on Sexual and Urinary Functions After Fully Robotic Nerve-Sparing Total Mesorectal Excision for Rectal Cancer. Annals of Surgery. 2013, 257(4): 672-678.

13. Emma R. McGlone, Omar A. Khan, John Conti, Zafar Iqbal, Amjad Parvaiz. Functional outcomes following laparoscopic and open rectal resection for cancer. International Journal of Surgery 2012, 10(6): 305-309.

14. Liu Ly, Liu WH, Cao YK, Zhang L, Wang PH, Tang LJ. Urinary Function following Laparoscopic Lymphadenectomy for Male Rectal Cancer. PLoS ONE 2013, 8(11): e78701.

15. 卫洪波, 王吉甫, 张维麟. 直肠癌根治术后尿流动力学变化的研究. 癌症. 1993, 12(5): 422-425.

16. 卫洪波, 朱天伦, 冯笑山. 直肠癌根治术后性功能障碍及其预防. 当代肿瘤学杂志. 1994, 3: 227-228.

17. 卫洪波, 王吉甫, 张维麟. 直肠癌根治术后性功能障碍的研究. 中国肛肠病杂志. 1998, 18(10):16-18.

18. 郑宗珩, 卫洪波, 陈图锋, 黄江龙, 魏波, 胡宝光, 郑峰, 郭卫平, 黄勇, 司徒杰. 盆腔自主神经保护的腹腔镜直肠癌根治术对患者排尿功能的影响. 中华医学杂志. 2009, 89(42): 2976-2979.

19. 黄江龙, 郑宗珩, 卫洪波, 方佳峰, 张实, 陈羽青. 盆腔自主神经活体尸体比对研究. 中华外科杂志. 2014, 52(7): 1-5.

20. 黄江龙, 郑宗珩, 卫洪波, 方佳峰, 张实, 陈羽青.直肠系膜结构解剖和腔镜下观察的对比研究. 中山大学学报. 2014, 35(3): 407-411.

21. Kim JH, Kinugasa Y, Hwang SE, Murakami G, Rodríguez-Vázquez JF, Cho BH. Denonvilliers' fascia revisited. Surg Radiol Anat. 2015 Mar;37(2):187-97.

22. M. M. Bertrand, B. Alsaid, S. Droupy, G. Benoit, M. Prudhomme. Optimal plane for nerve sparing total mesorectal excision, immunohistological study and 3D reconstruction: an embryological study. Colorectal Disease 2013, 15: 1521–1528.

23. Heald RJ, Moran BJ, Brown G, Daniels IR. Optimal total mesorectal excision for rectal cancer is by dissection in front of Denonvilliers' fascia. Br J Surg. 2004 Jan;91(1):121-3.

24. Kinugasa Y1, Murakami G, Uchimoto K, Takenaka A, Yajima T, Sugihara K. Operating behind Denonvilliers' fascia for reliable preservation of urogenital autonomic nerves in total mesorectal excision: a histologic study using cadaveric specimens, including a surgical experiment using fresh cadaveric models. Dis Colon Rectum. 2006 Jul;49(7):1024-32.

25. Liang JT. Lai HS, Cheng KW. Laparoscopic dissection of Denonvilliers' fascia and implications for total mesorectal excision for treatment of rectal cancer. Surg Endosc. 2011, 25: 935–940.

26. 卫洪波, 黄江龙, 郑宗珩, 魏波, 方佳峰, 黄勇, 陈图锋, 刘健培. 腹腔镜直肠癌根治术中保留 Denonvilliers 筋膜对男性排尿及性功能的影响. 中华胃肠外科杂志. 2015, 18(3): 82-87.

2. RESEARCH OBJECTIVE

The incidence of urinary and sexual dysfunction after TME for middle and low rectal cancer is high and affects the patients' quality of life. In recent years, scholars have improved the surgical methods to protect the pelvic autonomic nerve. Consensus was reached on the protection of the superior hypogastric plexus, hypogastric nerve and the origin of the inferior hypogastric plexus, and the above-mentioned urogenital dysfunction was reduced to some extent. However, how to best protect the descending branches of the hypogastric plexus remains controversial. Most surgeons still follow the "holy plane" of the classic TME in front of DVF, which is prone to vascular nerve bundle damage, and the end result is that urination and sexual dysfunction cannot be improved. Based on previous anatomical studies and preliminary clinical trials, we propose that TME surgery should be separated from the rear of DVF to keep the intact.

The objective of this study was to evaluate the efficacy of preserving or not preserving DVF in laparoscopic total mesorectal excision for urination and

sexual function protection in male patients with T1-4 (anterior wall T1-2), N0-2, M0 middle and low rectal cancer, and to evaluate the surgical safety and oncological safety of the two methods.

3. RESEARCH DESIGN

A prospective, multicenter, randomized design was adopted with open, positive parallel control, and validation of superiority. The research design meets ethical requirements.

3.1 Multicenter:

There are 11 centers participating in this research project. The research institutes include: 1) The Third Affiliated Hospital, Sun Yat-sen University; 2) Shantou Central Hospital; 3) Affiliated Hospital of GuangDong Medical University; 4) Anyang cancer Hospital; 5) The Sixth Affiliated Hospital, Sun Yat-sen University; 6) Nanfang Hospital, Southern Medical University; 7) Affiliated Hospital of Henan university of science and technology; 8) The Second Affiliated Hospital of Guangzhou Medical University; 9. Shunde Hospital, Southern Medical University; 10) The Second Affiliated Hospital, Sun Yat-sen University; 11) The First Affiliated Hospital, Xiamen University. And the statistics related issues are handled by the School of Public Health of Sun Yat-Sen University.

3.2 Grouping

Experimental group (Exp-group): DVF preservation during laparoscopic TME (L-TME); Control group (Con-group): DVF resection during L-TME.

3.3 Sample size determination

In this study, the incidence of urinary dysfunction 2 weeks after the operation and sexual dysfunction 12 months after the operation were the main measures of effectiveness. Our previous results showed that the incidence of urinary dysfunction was 24.39% at 2 weeks after DVFP-TME operation and the incidence of sexual dysfunction was 9.76% at 12 months after operation, compared to the corresponding incidence for L-DVFR-TME, which was 44.68% and 42.55%, respectively.

This study hypothesizes that the incidence of urinary dysfunction at 2 weeks and sexual dysfunction at 12 months after operation in the experimental group is the same as that in the control group. In the method of determining the sample size, the first two indicators use a one-sided test, setting the alpha value to 5%, and using 0.025 as the unilateral statistical significance level, setting the test efficiency to 90%, using a balanced design. The computational formula of unilateral test is as follows:

$$n_1 = \frac{[Z_\alpha\sqrt{p(1-p)(1+c)/c} + Z_\beta\sqrt{p_1(1-p_1)+p_2(1-p_2)/c}]^2}{(p_1-p_2)^2}$$

The largest sample size was obtained by unilateral urinary dysfunction test, which required 110samples for each group. Considering that the largest abscission rate in this clinical study was about 10%, the final sample size of each group was 121, and the total number of cases needed was 242. An open, competitive approach was adopted for case enrollment in each center. According to the established research plan, the preliminary functional evaluation results will be reported after enrollment is completed.

3.4 Randomization

A central stratification and block group randomization was used. The stratification factor was center and the block size was 4. Given the number of seeds and the length of segments, SAS 9.2 (SAS Institute, Cary, NC) was used to generate the treatment allocation corresponding to the flow number 001-242. During the research process, if a patient withdraws from the research, the corresponding random sequence needs to be expanded. Case enrollment is reviewed by the surgeon according to the inclusion and exclusion criteria. After the case is selected, the information of enrollment will be sent to the central randomization department, which will analyze the case information and determine the grouping, and then inform the research center. Considering the different progress of each center, the random number is provided in segments.

3.5 Blind method

A single-blind design is adopted in this study. The case enrollment information is managed by the central randomization department. When the patient is included in the study, the research assistant sends the enrollment information to the central randomization department. After the group is determined by the central randomization department, the research center is informed before the operation. The participants and research assistants who participated in patient follow-up and functional evaluation were blinded.

3.6 Research cycle

Case enrollment cycle: all centers completed case enrollment within 4 years.

Follow-up period: The first case was included as the starting point of follow-up, and the last case was included as the follow-up endpoint of the main study for the next 3-5 years. The research progress is shown in the following table:

Annual Planning Arrangements	
**Total duration of project implementation**	**7 years**
**Start and stop time**	**Main Contents and Achievements of Phase Objectives**
**2015.7-2015.8**	1. Formulate and improve the entire set of project implementation procedures and surgical specifications.<br>2. Qualification assessment of sub-centers.

	3. Conduct PI and research assistant training for each center.<br>3. Project preparatory meeting: to coordinate the division of tasks.<br>4. Prepare for the implementation, and start research work.
2015.8-2019.7	Case randomization and study implementation
2019.8-2019.12	Interim analysis of the clinical study, and determine whether to continue enrolling based on the results of the interim analysis.
2019.6-2019.12	Cases randomization, study performed, and follow-up of enrolled patients. The group was terminated as of December 2019.
2020.1-2024.12	Case follow-up and final results were announced.

4. RESEARCH OBJECTIVE

All patients who meet the inclusion criteria and do not belong to any exclusion criteria are eligible for this study.

4.1 Inclusion criteria:

- Male, 20 yrs < age < 71 yrs, informed consent;
- Pathological diagnosis of rectal adenocarcinoma;

- Tumors from anal edge 6 ~ 12 cm (measured by rigid proctoscope);
- Preoperative staging T1-4 (T1-2 for anterior rectal wall) N0-2M0 rectal cancer (AJCC - 7 th);
- R_0 TME surgical results are expected;
- Preoperative ECOG physical status score 0/1;
- Preoperative ASA grade I ~ III;
- With normal urinary function (bladder residual urine <100 ml); normal erection function (IIEF-5>21) and ejaculation function grading as I level.

4.2 Exclusion criteria:

- Complication with acute ileus, perforation or hemorrhage;
- Tumors with extensive invasion of surrounding tissues, TME not applicable;
- With neoadjuvant radiotherapy;
- Imaging examination in regional integration intumescent lymph nodes (maximum diameter 3 cm or greater);
- With other malignant diseases or with other malignant disease within 5 years;
- With other severe diseases needing surgery;
- A history of abdominal or pelvic major operation;
- People with severe mental illness, or that cannot be evaluated due to cultural or psychological factors;
- No sex life;

- Critical organ dysfunction, unbearable surgery;
- 6 months of unstable angina or a history of myocardial infarction and cerebral infarction or hemorrhage;
- 1 month on systemic corticosteroids or immunosuppressive medication history;
- Preoperative urinary incontinence or severe stress urinary incontinence.

4.3 Exit criteria:

- Confirmed as M1 during or after operation: no evidence of distant metastasis found during preoperative examination, and intraoperative exploration/postoperative pathology confirmed distant metastasis, or postoperative confirmed peritoneal lavage cytology was positive;
- Conversion to abdominoperineal resection (APR);
- Tumor infiltration beyond the rectal intrinsic fascia, or confirmed as T3 for anterior rectal wall;
- Intraoperative confirmed regional lymph node fusion conglobation cannot ensure R0 resection;
- Tumors wrap around important blood vessels without resectability;
- Other diseases found during surgery that require concurrent surgical treatment;
- Preoperative emergent severe complications; cannot undergo the study treatment;
- Condition changes, emergency operation is needed;

- Into this study, at any stage of the initiative exit or discontinue treatment;
- Prove to implement the treatment violates this research plan.

5. STUDY ENDPOINT

5.1 Primary study endpoint

- Incidence of urinary dysfunction 2 weeks after surgery
- Incidence of sexual dysfunction (erectile and ejaculation dysfunction) 12 months after surgery

5.2 Secondary study endpoint

- 1,3-year overall survival rate
- 1,3-year disease free survival rate
- 1,3-year recurrence and recurrence pattern
- Morbidity and mortality (30 days postoperatively) rates
- Postoperative recovery course

6. DIAGNOSTIC CRITERIA FOR THIS STUDY

(1) AJCC-7th TNM staging system was used in this study.

(2) Primary rectal lesions were diagnosed as rectal adenocarcinoma by endoscopic biopsy (tubular adenocarcinoma, papillary adenocarcinoma, mucinous adenocarcinoma, signet ring cell carcinoma).

(3) Definition of mid-low rectal cancer: the distance between the tumor and anal margin is less than 12 cm for mid-low rectal cancer.

7. QUALIFICATION OF RESEARCHERS

7.1 Basic principle

The surgeons responsible for this study must meet the following conditions: (1) must have completed more than 50 cases of laparoscopic radical resection of rectal cancer; (2) blind examination through surgical video.

7.2 Specific measures

(1) The number of completed cases in the past shall be certified in writing by the case Department of each applicant unit.

(2) Blind examination of surgical video : three consecutive video clips of laparoscopic radical resection of rectal cancer were provided by the applicant to the project research committee in the past month. Two cases were randomly selected by the Research Committee for blind review. If the experts of the research committee unanimously approved the video-displayed surgical techniques and the degree of cancer eradication, the applicant was allowed to join the study as a researcher.

8. STANDARDIZED OPERATION PROCESS

8.1 Case selection process

8.1.1 Assessment items selected by patients

- General condition: ECOG score, height, weight, BMI, vital signs;
- Laboratory examination: Hb, RBC, WBC, LYM, NEU, NEU%, PLT;
- Blood biochemistry: albumin, prealbumin, total bilirubin, AST, ALT, creatinine, urea nitrogen, fasting blood sugar, CRP;
- Serum Oncological Indicators: CEA、CA199;
- Enteroscopy and histopathology:
- Abdominal spiral plain and enhancement CT scan: thickness of the layer less than 10 mm.
- Pelvic MR or transrectal ultrasound;
- Positive and lateral radiographs of the chest;
- ECG
- Respiratory function test (FEV1、FVC): depends on the patient's condition.
- Evaluation of urinary function: residual urine of bladder (evaluation by ultrasound), maximum urinary flow rate (urodynamics), International Prostate Symptom Score (i-PSS);
- Sexual function status [erectile function was evaluated by IIEF-5, ejaculation function was graded according to Ejaculation Grading (grade I was ejaculation, ejaculation quantity was normal or decreased, ejaculation function was normal; grade II was retrograde ejaculation, ejaculation dysfunction; grade III was complete non-ejaculation).

8.1.2 Application for inclusion

The details of patients who meet the inclusion criteria and do not fall into the exclusion criteria are faxed to the research committee by the research assistants of each center before the study, and the latter examines whether they are eligible for admission.

8.1.3 Relevant Counseling on Enrollment Qualifications

- Dr. Bo Wei：13527794069
- Dr. Purun Lei：18620121924

8.1.4 Attention

- The eligibility application must be completed before the operation; application after operation will not be accepted.
- The application form for eligibility must be adequate (including name, age, identity card number, hospital number).
- Pathological type and preoperative staging;
- Each center's accreditation notice shall be kept and filed by the research assistant.
- Once selected, the eligibility is not allowed to be artificially cancelled unless the patient refuses to use the information for this study.
- Data centers reject any duplicate information. In case of duplicate information, first registered information will be used;
- In case of duplication and misregistration, the research assistant should

contact the Research Committee as soon as possible

8.2 Preoperative management

Operations should be performed within one week after eligibility has been obtained.

- **Preoperative evaluation: 1)** Evaluation of urinary function: residual urine of the bladder (evaluation by ultrasound), urodynamics (maximum urinary flow rate), International Prostate Symptom Score (i-PSS). 2) Sexual function status [erectile function was evaluated by using IIEF-5, ejaculation function was graded according to ejaculation function grading (grade I was ejaculation, ejaculation quantity was normal or decreased, ejaculation function was normal; grade II was retrograde ejaculation, ejaculation dysfunction; grade III was complete non-ejaculation). 3) Preoperative systemic organ function evaluation included cardiac function, respiratory function, renal function, liver function and nervous system function; other preoperative evaluation indicators included tumor staging (preoperative staging based on chest X-ray, abdominal CT, pelvic MR or intracavitary ultrasound), ECOG physical state score, ASA score, biochemical (CRP, AST, ALT, TBLI, BUN, Cr and GLU), blood routine, tumor markers (CEA, CA199).
- Emergency surgery should be excluded if the clinical condition deteriorates from the time of selection to the expected operation day.

- Preoperative enteral or parenteral nutrition support is allowed for patients at nutritional risk.
- For basic diseases such as cardiopulmonary, cerebral and metabolic diseases, they should be evaluated and preoperatively prepared according to medical specialist's opinions, and should be recorded in CRF.
- Laparoscopic total mesorectal excision (L-DVFP-TME or L-DVFR-TME) with preservation and removal of DVF was used in the choice of operative methods. Specifications regarding whether reconstruction or anastomosis of the digestive tract was performed were not required according to the central habits; whether preventive ileostomy was needed or not was not required.
- Preoperative fasting and water fasting were performed according to the routine anesthesia protocol of each center.
- Preventive use of antibiotics: the first dose should be given 30 minutes before the operation, and an additional dose should be added at least 3 hours after the operation.
- Preoperative data collection: as mentioned above.

8.3 Random Grouping

- A central stratification and block group randomization was used. Given the number of seeds and the length of segments, SAS 9.2 was used to generate the treatment allocation corresponding to the flow number 001-242.

- After the case is selected, enrollment information will be sent to the central randomization department, which will analyze the case information, determine the grouping and then inform the research center. Considering the different progress of each center, the random number is provided in segments. All research centers strictly follow the grouping and operation criteria.

Central Randomization Departments Contacts

- Prof. Jinxin Zhang: 13660501365
- Prof. Yunlian Xue: 13631351625

8.4 Standardization of surgical procedures

8.4.1 Common principles

- **Anesthesia:** general anesthesia with tracheal intubation.
- **Pneumoperitoneum:** carbon dioxide pneumoperitoneum was used to maintain pressure of 12-13 mmHg.
- **Provisions for puncture or auxiliary incision:** location is not specified, no more than 5 punctures, only one auxiliary incision with a length of less than 10 cm.
- **Definition of laparoscopic approach:** laparoscopic instruments must be used to perform intraperitoneal operations with the support of a video camera system. Rectal and mesenteric free, lymph node dissection, rectal amputation, and submesenteric arteriovenous amputation are all performed under laparoscopy. Rectal resection allows the use of an

auxiliary small incision in the open state, and anastomosis must be performed under laparoscopy; for example, combined abdominal and perineal resection or non-anastomosis. It should be clearly recorded in the CRF.

- **Intraoperative exploration:** to explore the abdominal cavity comprehensively and find out whether the liver and other parenchymal organs have metastasis. To explore whether the surface of cavity organs and omentum mesentery, and peritoneum have implantation metastasis or not.
- **Regulations for rectal resection:** low rectal anterior resection was performed according to the " Criteria for diagnosis and treatment of colorectal cancer (2010)" and " Surgical clinical pathway for Low Anterior Rectal Cancer" of the Ministry of Health in China, and NCCN Guidelines for the Treatment of Colorectal Cancer (2014 edition). The distal resection margin of the rectum is at least 2 cm away from the tumor, and the proximal resection margin usually exceeds 10 cm.
- **Scope of mesorectal excision:** the visceral fascia and the surrounding rectal fat, blood vessels and lymphatic vessels, the so-called mesorectal, are totally removed.
- **Scope of lymph node dissection:** the lymph nodes in the mesorectal region can be removed by TME, and the corresponding lymph nodes in the mesorectal region can be removed by ligation of the root of the inferior

mesenteric artery. If there is no indication of preoperative imaging data or no obvious enlarged lymph nodes found during intraoperative exploration, lateral lymph node dissection is not required.

- In order to preserve pelvic autonomic nervous function, PANP should be performed according to the following requirements:

1) Abdominal aortic plexus can be easily injured when ligating the inferior mesenteric artery. When pulling the sigmoid colon upward, the distance between the superior mesenteric artery and abdominal aortic plexus becomes larger and 1-1.5 cm from the root of the inferior mesenteric artery is ligated to avoid injury to the abdominal aortic plexus.

2) Hypogastric nerve protection: when the posterior rectum is free, the smooth mesorectum of the rectum should be close to the top of the loose reticular structure, and the sharp separation should be made downward to avoid damaging the superior hypogastric plexus and left and right hypogastric nerves below the loose reticular structure.

3) Protection of the hypogastric plexus: when the lateral ligament is cut off, the rectal ligament should be cut in the medial part of the vascular nerve bundle to avoid injuring the lateral hypoventral plexus without causing bleeding.

4) Protection of the efferent branch of the hypogastric plexus: the main difference between the two groups was whether or not DVF was preserved.

Exp-group: lateral DVF, close to the seminal vesicle and the outer margin of the prostate, can be seen from the inferior hypogastric plexus of the seminal vesicle and prostate innervation nerve traveling here, as well as the seminal vessel and prostate trophic vessels. When the anterior part of the lower rectum is free, the pelvic floor peritoneum should be incised at the lowest point of peritoneal retraction, and the integrity of the seminal vesicle capsule and DVF (i.e. sharp separation of the anterior rectal space) should be protected, and the mesorectal membrane should be free under the loose reticular structure.

Con-group: the pelvic floor peritoneum was incised 1 cm above the peritoneal reentry. The anterior rectum was dissected through DVF (i.e. the posterior prostate space) and DVF was excised.

- **Regulations for digestive tract reconstruction:** each sub-center can choose the anastomosis method in accordance with their own technique , without detailed stipulation, and usually an end-to-end anastomosis.
- **Provisions for conversion to open surgery:** intra-abdominal hemorrhage and organ damage caused as a result of surgery are serious/dangerous. When the life of the patient is in danger and the surgical complication is difficult to control under laparoscopic condition, it is necessary to actively switch to a laparotomy. The occurrence of carbon dioxide pneumoperitoneum also requires a switch to laparotomy. Other technical

and instrumental factors can also lead to conversion to laparotomy, a switch which is decided by the surgeon responsible for the operation. The length of incision for conversion to laparotomy is not regulated in this study. According to the Intentional Therapy (ITT) principle, cases transferred to laparotomy are still regarded as laparoscopic cases and analyzed in PP. The reasons for conversion to open surgery should be clearly recorded in the CRF.

- **Follow-up management of cases excluded in laparoscopic group:** Continuation of laparoscopic surgery or conversion to laparotomy is decided by the surgeon responsible for the operation, who can decide based on his/her own clinical experience.
- **Regulations on equipment and instruments related to surgery:** the equipment and surgery instruments will be chosen according to the techniques of each center.
- **Gastric tube and drainage tube:** usually without indwelling gastric tube or after removal of gastric tube, abdominal drainage tube indwelling 1 in the pelvic floor.
- **Provisions for the implementation of other operations at the same time:** if other diseases are found during the operation and need to be operated on at the same time, the case should be excluded.
- **Image/Photo Recording:** Submesenteric artery ligation (1 photo), bilateral inferior ventral nerve (1 photo), bilateral rectal lateral ligament (1 photo

each), anterior rectal wall and DVF (1 photo), resection of the general rectal specimen appearance (from the anterior wall showing mesentery, 1 photo), rectal section showing tumors (1 photo) and distal and proximal incision (1 photo, scaling), skin incision (1 photo, scaling); Video recordings of laparoscopic surgery should be kept for reference.

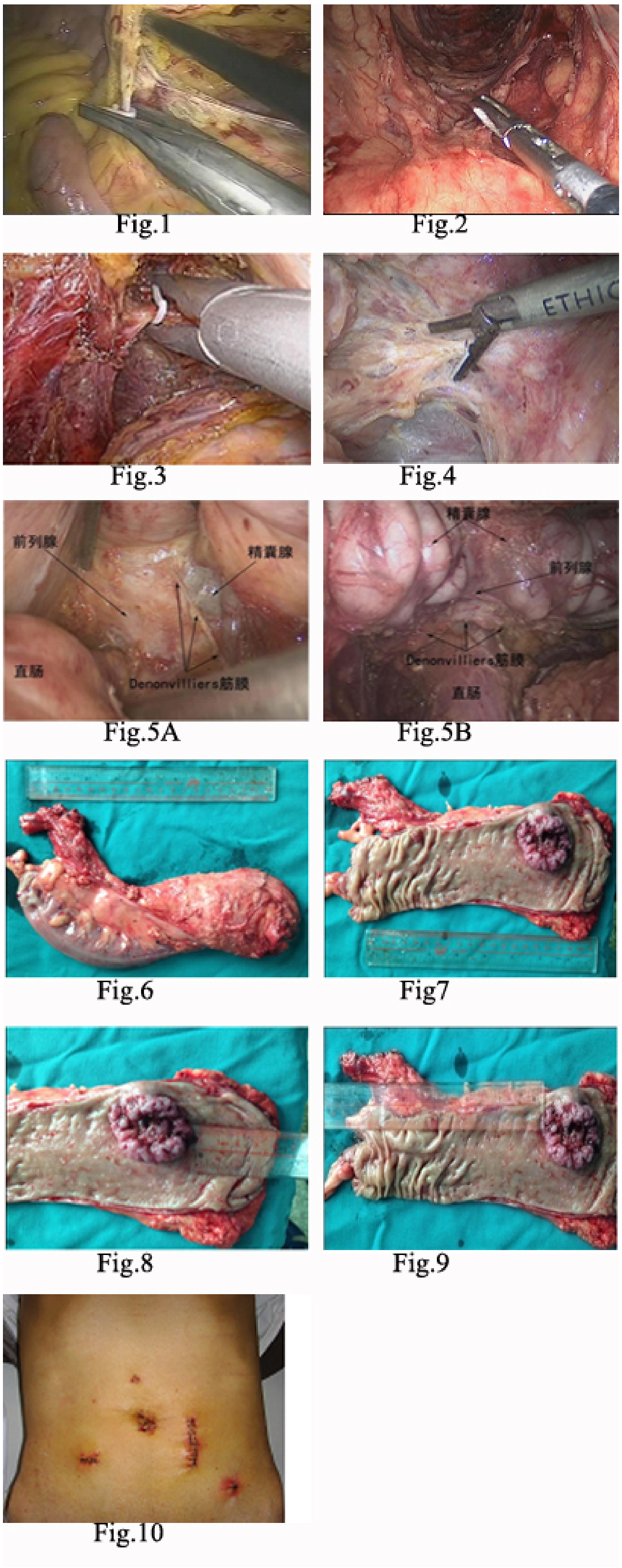

Fig 1. Submesenteric artery ligation;

Fig 2. Bilateral inferior ventral nerve;

Fig 3 and Fig 4. bilateral rectal lateral ligament; Fig 5A. and Fig 5B. anterior rectal wall and DVF; Fig 6. Excised gross specimen; Fig 7. rectal section showing tumors; Fig 8 and 9. distal and proximal incision (1 piece, scaling); Fig 10. skin incision.

- Photo data protection: photographic data should never reveal the patient's identity, of affect their personal privacy, and each patient's photo should be placed in the same folder, folder name as : [L-DVFP-subject number] / [L-DVFR-subject number]; photo is named as" [Fig. + serial number + part name], for example: [Fig .1 inferior mesenteric artery ligation] / [Fig. 5 anterior rectal wall and DVF].
- Image data storage: all photographic data are stored in digital format on a hard disk or mobile digital carrier, and should be uploaded within 1 week after surgery by "Baidu cloud storage". Videos from each research center are independently backed up; the video of laparoscopic surgery is saved by the participating research institutes themselves.
- Evaluation of surgery: surgical indication, lymphadenectomy quality, auxiliary incision length and specimen completeness (CRM quality) were evaluated by the research committee based on the surgical video and photos. Video recordings of laparoscopic surgery were performed and uncut image files were kept. The Research Committee reviewed and monitored the quality of laparoscopic surgery through

the above channels.

8.4.2 Surgical outcomes

- Surgeon's information;
- Surgical duration;
- Lymphadenectomy range and digestive tract reconstruction method;
- Conversion to laparotomy surgery and reason;
- Estimated blood loss;
- Blood transfusion;
- Tumor location (anterior/posterior/lateral wall);
- Tumor size (maximal diameter, mm);
- Status of circumferential resection margin, invasion depth, tumor occupation, harvested lymph node number, distant metastasis;
- Length of proximal and distal margin (mm), radical degree （R0/R1/R2）;
- Intra-operative morbidity (occurs between the beginning of peeling and the completion of seaming): intraoperative bleeding and injury (important organs and structures, including extra blood loss due to vascular injury); pneumoperitoneum related complications, including hypercapnia, mediastinal emphysema, subcutaneous emphysema, air embolism, and respiratory cycle instability caused by pneumoperitoneum. Anesthesia related complications.
- Intraoperative death (during the start of the incision to the

completion of the suture): for whatever reason.

8.4.3 Postoperative Pain Management

- Prophylactic application of painkillers: postoperative continuous intravenous analgesia is usually prescribed within 48 hours of surgery. The dose, type, and infusion rate are administered by the anesthesiologist according to clinical routine and patient specific conditions. Preventive analgesics should not be reused after 48 hours of surgery unless clinically determined.
- Postoperative rehydration and nutritional support: postoperative rehydration (including glucose, insulin, electrolytes, and vitamins) or nutritional support (intestinal / parenteral), according to the experience of the competent doctor and clinical routine, this study does not make provisions. After oral ingestion, fluid/nutrition support will be stopped or gradually reduced.
- Postoperative rehabilitation management: the management of the incision, gastric tube, and abdominal drainage tube is routinely performed according to the diagnosis and treatment operation. The recovery of eating time and the dietary transition strategy are routinely performed according to the diagnosis and treatment operation principles.
- Perioperative surgical outcomes: operation time, EBL, specimen observation [circumferential margin (surgical doctor to carry out

circumferential margin integrity quality rating, and to take 1 mm circumferential margin), tumor size, tumor occupation, proximal and distal margin, number of lymph nodes harvested and stage, perioperative complication rate [including systemic complications (lung Infection, pneumothorax, hypercapnia, deep vein thrombosis, myocardial infarction, heart failure, renal failure, liver failure), surgical site infection, abdominal infection, digestive tract infection, anastomotic leakage, anastomotic bleeding, postoperative ileus (anatomic obstruction, intestinal obstruction), ostomy complications (such as preventive ileostomy, including stoma hemorrhage, stoma necrosis, stoma infection, parastomal hernia, stoma retraction, ostomy prolapse and stenosis), postoperative gastrointestinal function recovery time (recovering exhaust time, time to having fluids and solid diet) and hospitalization days.

8.4.4 Patient discharge criteria

No postoperative complications were found, and "tolerable oral half-flow food" and "routinely exercise" were met, and the discharge was arranged, and recorded in the CRF.

8.4.5 Postoperative outcomes

- Pathological results: histological type, depth of invasion, distant metastasis and location, histological grade, radical degree (R0/R1/R2) , harvested number of lymph nodes, positive lymph nodes, proximal

/distal margins and the circumferential margin status.

- Early postoperative complications (within 30 days after surgery): incision complications (infection, effusion, dehiscence, poor healing, etc.), formation of peritoneal effusion or abscess, intraperitoneal active hemorrhage, gastrointestinal active hemorrhage, intestinal obstruction, intestinal paralysis, anastomotic stenosis, anastomotic fistula, intestinal fistula, lymphatic leakage, deep venous thrombosis, pneumonia, urinary tract infection, renal failure, liver failure, cardio-cerebrovascular events (including thrombosis) (thrombus, embolism, etc.)
- Blood test (on 1st, 3rd, 5th day): peripheral blood routine test for : Hb、RBC、WBC、LYM， NEU、NEU%、PLT； blood biochemistry: albumin, prealbumin, total bilirubin, AST, ALT, creatinine, urea nitrogen, fasting blood glucose, CRP.
- Postoperative rehabilitation evaluation: flatus/defecation time (hour), recovery of oral intake (hour), postoperative to discharge daily maximum body temperature (°C), removal of abdominal drainage tube time (d), daily drainage (M), transfusion (ml) and postoperative LOS (d) after surgery.
- Postoperative clinical pathological staging: according to pathological results (including tumor location, pathological type, degree of differentiation, circumferential margin, proximal and distal margins

and lymph node metastasis), as well as preoperative clinical data, to obtain accurate clinical pathology staging.

- Long-term postoperative complications (more than 30 days after surgery to 3 years after surgery): incision/puncture hernia, anastomotic stenosis, mechanical intestinal obstruction, stoma-related complications (stoma stenosis, stoma retraction, parastomal hernia, etc).
- Postoperative urinary function: ultrasound examination of bladder residual urine, urodynamics, maximum urinary flow rate and prostate symptom scores was performed 14 days, 1 month, 3 months and 6 months after surgery.
- Postoperative sexual function status evaluation: follow-up after 1 month, 3 months, 6 months, 12 months, 18 months and 24 months after surgery. Follow-up can be performed by questionnaires, telephone, etc. Record the reduction in postoperative work ability, emotional changes in the spouse, and loss of libido, and erectile dysfunction was evaluated according to the International Erectile Function Questionnaire-5 (IIEF-5); the ejaculation function was evaluated by ejaculation function grading.

8.5 Follow-up

8.5.1 Follow-up cycle and precautions

- Each research center arranged a follow-up commissioner to follow up all the cases enrolled in the study. Patients were followed up every 3 months within 1 year after surgery; 1 year later, every 6 months (ie, 3 months, 6 months, 9 months, 12 month , 18 months, 24 months, 30 months, 36 months, 42 months, 48 months, 54 months, and 60 months after surgery).
- This study suggests that the above examinations should be conducted at the research center where the patient underwent surgery, although other hospitals are also allowed. It is recommended that the hospital be a third-grade hospital, and the follow-up commissioner will track and record the results of each inspection.
- Researchers should evaluate the results of each examination, record the postoperative survival status of all patients, and define tumor recurrence or metastasis.
- Patients refusing to follow up according to the above mentioned plan, they are recorded as lost cases. At the end of the study, they are analyzed together with the cases that meet the research criteria (the PP set is not removed).

8.5.2 Examinations during follow-up

- Full physical examination: performed by the competent doctor at regular follow-up, record the patient's quality of life score (QOL),

ECOG physical status score, eating and defecation after surgery, general condition, abdominal signs and digital rectal examination.

- Urinary function follow up in 1 year after surgery is shown in 8.4.5.
- Postoperative sexual function evaluation is shown in 8.4.5.
- Blood test: peripheral blood routine, blood biochemistry (CRP, AST, ALT, TBLI, BUN, Cr and GLU), serum tumor markers (CEA, CA199).
- Imaging examination items: abdominal CT scan (including pelvic cavity) (layer thickness less than 10 mm; when the contrast agent is allergic, only CT scan or MRI is allowed); digestive tract endoscopy (pathological histological biopsy if necessary, endoscopic ultrasound); chest radiograph (positive lateral position, chest CT if necessary); other assessment methods: gastrointestinal angiography, other parts of ultrasound, whole body bone scan, PET-CT, etc., when the competent doctor thinks it is necessary. Regular follow-up was performed and the 1-year, 3-year, and 5-year survival rates, local tumor recurrence rate, and other types of recurrence were recorded.

8.6 Postoperative adjuvant therapy

8.6.1 Indications for postoperative adjuvant chemotherapy

- According to the postoperative staging of patients, adjuvant therapy should be used for patients with stage II and III, and mFOLFOX6 and XELOX should be recommended for adjuvant chemotherapy.

- The specific implementation of adjuvant chemoradiotherapy is not mandatory, but it should be guaranteed that the two groups are equivalent.

8.6.2 Safety evaluation of postoperative adjuvant chemoradiotherapy

- Physical strength status (ECOG).
- Subjective and objective condition (according to CTCAE v3.0 records).
- Blood test.
- Safety evaluation items implemented when necessary in chemotherapy: neurotoxicity, cardiovascular system (myocardial toxicity, ischemic heart disease, etc.), infection due to bone marrow suppression and immune dysfunction, and other adverse reactions.

8.7 Study calendar

The project has a research process checklist to allow the researcher to easily check guideline implementation, to ensure the quality of the research and to prevent the research results from being lost or not recorded. The process monitoring table is as follows:

8.7.1 Screening and perioperative period

	Interview 1	Interview 2	Interview 3（POD）							
	pre-operatio	operation	1	2	3	4	5	6	14	hospital discharge

	n									
Actual follow-up date										
Demographic data	√									
Anamnesis	√									
Physical examination	√									
Operation information		√								
Specimen measurement		√								
Postoperative recovery			√	√	√	√	√	√	√	√
Postoperative physiological index			√		√		√		√	
ECOG	√									
ASA	√									
Blood routine examination	√		√		√		√		√	
Blood biochemistry	√		√		√		√		√	
Tumor marker	√									
CRP	√		√		√		√		√	
ECG	√									
Respiratory function	√									
Chest radiography	√									
Colonoscopy	√									
Pelvic MR										

Abdomen CT	√									
Pathological examination	√						√			
Urodynamics	√								√	
IPSS	√								√	
IIEF-5	√									
Ejaculation Grading	√									
Intraoperative and postoperative complications		√	√	√	√	√	√	√	√	√

8.7.1 Follow-up period

Interview	4	5	6	7	8	9	10	11	12	13	14	15
PoM	1	3	6	12	18	24	30	36	42	48	54	60
Follow-up date												
PE	√	√	√	√	√	√	√	√	√	√	√	√
BRT	√	√	√	√	√	√	√	√	√	√	√	√
Biochemistry	√	√	√	√	√	√	√	√	√	√	√	√
Tumor markers	√	√	√	√	√	√	√	√	√	√	√	√

Chest X-ray	√	√	√	√	√	√	√	√	√	√	√	√
Endoscope				√		√		√		√		√
CT			√	√	√	√	√	√	√	√	√	√
Chemotherapy	√	√	√									
Urodynamics	√	√	√	√	√	√						
IPSS	√	√	√	√	√	√	√	√	√	√	√	√
IIEF-5		√	√	√	√	√	√	√	√	√	√	√
Ejaculation Grading		√	√	√	√	√	√	√	√	√	√	√
Ability to work	√	√	√	√	√	√	√	√	√	√	√	√
Marriage bonds		√	√	√	√	√	√	√	√	√	√	√

The relevant ECOG physical status score, ASA score, erectile function according to the International Erectile Function Questionnaire-5 (IIEF-5)

score, the tumor patient's quality of life score (QOL) and the International Prostate Symptom Score (I-PSS) are shown in 8.8.

8.8 SOP definition

8.8.1 ECOG performance status

- **grade 0**: fully active, able to carry out all pre-disease activities without restriction.
- **grade 1**: restricted in physically strenuous activity but ambulatory and able to carry out work of a light or sedentary nature, e.g., light housework, office work.
- **grade 2:** ambulatory and capable of all self-care activities but unable to carry out any work activities, up and about more than 50% of waking hours.
- **grade 3:** capable of only limited self-care, confined to bed or chair more than 50% of waking hours.
- **grade 4:** completely disabled, cannot carry out any self-care, totally confined to a bed or chair.
- **grade 5:** dead.

8.8.2 ASA classifications from the American Society of Anesthesiologists

- Grade I: normal healthy patient
- Grade II: patients with mild systemic disease, no functional limitations,

has a well-controlled disease of one body system.

- Grade III: patients with severe systemic disease, with some functional limitation, has a controlled disease of more than one body system or one major system, no immediate danger of death.
- Grade IV : patients with severe systemic disease that is a constant threat to life. Has at least one severe disease that is poorly controlled or at end stage, possible risk of death.
- Grade V: moribund patients who are not expected to survive without the operation.
- Not expected to survive > 24 hours without surgery, imminent risk of death.

8.8.3 Quality of Life Score (QOL) for cancer patients

In 1990, China developed a draft with reference to foreign indicators, the criteria of which are as follows (scores are in brackets):

- Appetite: 1 can hardly eat; 2 food intake < normal 1/2; 3 food intake is normal 1/2; 4 food consumption is slightly less; 5 food intake is normal.
- Spirit: 1 is very poor; 2 is poor; 3 has an effect, but good and bad; 4 is still good; 5 is normal, the same as before the disease.
- Sleep: 1 difficult to sleep; 2 sleep is poor; 3 poor sleep; 4 sleep slightly worse; 5 is generally normal.
- Fatigue: 1 often tired; 2 consciously weak; 3 sometimes tired; 4

sometimes mild fatigue; 5 no fatigue.

- Pain: 1 severe pain with passive position or pain for more than 6 months; 2 severe pain; 3 moderate pain; 4 mild pain; 5 no pain.
- Family understanding and cooperation: 1 completely not understanding; 2 poor; 3 general; 4 family understanding and care is better; 5 good.
- Colleagues' understanding and cooperation (including leadership): 1 full understanding, no care; 2 poor; 3 general; 4 minority understanding care; 5 most people understand and care.
- My own understanding of cancer: 1 disappointment, does not cooperate at all; 2 uneasy, barely cooperate; 3 uneasy in general; 4 uneasy, but can cooperate better; 5 optimistic, confident.
- Attitude towards treatment: 1 does not have hope for treatment; 2 is dubious about treatment; 3 wants to see curative effect, and is afraid of side effects; 4 hopes to see curative effect, can still cooperate; 5 confident, active cooperation.
- Daily life: 1 bed; 2 can be active, more than half of the time in bed; 3 can move, sometimes bed; 4 normal life, cannot work; 5 normal life work.
- Side effects of treatment: 1 seriously affects daily life; 2 affects daily life; 3 after symptomatic treatment can not affect daily life; 4 asymptomatic treatment can not affect daily life; 5 does not affect daily life.
- Facial expression: 1 to 5 levels.

The current quality of life grading: cut off score is 60 points, very poor <20 points, mild poor is 21~30.

8.8.4 International Prostate Symptom Score Sheet (I-PSS)

Have you experienced any of the following symptoms in the past 1 month？	No	Less than 1 out of 5	Fewer Than Half	About half	More than half	Nearly every time	Symptom score
1. Often feeling of needing to urinate？	0	1	2	3	4	5	
2. Is the interval between urinations often less than two hours?	0	1	2	3	4	5	
3. Is intermittent urination is frequent？	0	1	2	3	4	5	
4. Do you often have difficulty holding your urine？	0	1	2	3	4	5	
5. Do you often experience the phenomenon of urinary line thinning？	0	1	2	3	4	5	
6. Does it often require an effort and exertion to start urinating？	No	Once	Twice	Three times	Four times	Five times	
	0	1	2	3	4	5	
7. From going to sleep to waking up early do you generally need to urinate a few times？	0	1	2	3	4	5	
Total Symptom score =							

Note：0-7：Normal - mild symptoms；8-19：Moderate prostate symptoms；20-35：Severe prostate symptoms

8.8.5 Erectile function according to the International Erectile Function Questionnaire-5 (IIEF-5)

Please answer the following questions based on your actual sex life in the past 6 months （√）

	0	1	2	3	4	5	Score
1. Confidence in the erection of the penis and its maintenance？		Very low	Low	Moderate	High	Very high	
2. How many times can the penis be firmly inserted into the vagina after sexual stimulation？	No sexual activities	Little or no	Only a few times	Sometimes or about half the time	Most of the time	Almost every time or every time	
3. How many times can sexual intercourse maintain penile erection after entering the vagina？	No sexual activities	Little or no	Only a few times	Sometimes or about half the time	Most of the time	Almost every time or every time	
4. How difficult is it to maintain an erection during intercourse until the intercourse is complete？	Not trying to have sex	Very difficult	Moderate difficulty	Difficult	A little difficult	Not difficult	
5. Do you feel satisfied when trying to have sex？	Not trying to have sex	Little or no	Only a few times	Sometimes or about half the time	Most of the time	Almost every time or every time	
**IIEF-5 Score：**							

Note：In general, an IIEF-5 score of less than 7 is classified as severe erectile dysfunction; 8-11 is classified as moderate erectile dysfunction; and 12-21 is classified as mild erectile dysfunction.

In this study, erectile dysfunction was defined as an IIEF-5 score of ≤11 points.

8.8.6 Ejaculation function is classified according to ejaculation function grading

- **Grade I** is ejaculation, the amount of ejaculation is normal or reduced, and the ejaculation function is normal.
- **Grade II** is retrograde ejaculation with ejaculation dysfunction.
- **Grade III** is complete ejaculation.

Note: Gradel I represents normal ejaculation while Grade II~III represent ejaculation dysfunction.

8.8.7 Oncology-related definitions

The tumor staging of this study was based on the TNM staging of AJCC-7.

8.8.7.1 Primary position

The position of the primary lesion defined in this study is based on the distance from the anal margin, and the rectal examination is the primary criterion. Colonoscopy and imaging examination results can be used for reference.

8.8.7.2 Recording tumor staging

Tumor staging is divided into clinical stage and pathological stage, involving T (rectal wall infiltration depth), N (regional lymph node), and M (distant metastasis), marked with capital letters; the degree is expressed by Arabic numerals, and when not clear Expressed by X. The clinical stage is mainly based on physical examination, X-ray, endoscopy, imaging diagnosis, intraoperative findings, biopsy cytology and biochemical examination. Pathological staging is based on pathological diagnosis of endoscopy and surgical specimens.

8.8.7.2.1 Primary tumor (T)

Tx: Primary tumor cannot be assessed

T0: No evidence of primary tumor

Tis: Carcinoma in situ intraepithelial or invasion of lamina propria

T1: Tumor invades submucosa

T2: Tumor invades muscularis propria

T3: Tumor invades through the muscularis

T4a: Tumor penetrates to the surface of the visceral peritoneum rectal tissues

T4b: Tumor directly invades or is adherent to other organs or structures

8.8.7.2.2 Regional Lymph Nodes(n)

Nx: Regional lymph nodes cannot be assessed

N0: No regional lymph node metastasis

N1: Metastasis in 1-3 regional lymph nodes

N1a: Metastasis in one regional lymph node

N1b: Metastasis in 2-3 regional lymph nodes

N1c: Tumor deposit(s)in the subserosa, mesentery, or nonperitonealize pericolic or perirectal tissues without regional nodal metastasis

N2: Metastasis in four or more regional lymph nodes

N2a: Metastasis in 4-6 regional lymph nodes

N2b: Metastasis in seven or more regional lymph nodes

8.8.7.2.3 Distant Metastasis(M)

M0: No distant metastasis

M1: Distant metastasis

M1a: Metastasis confined to one organ (eg, liver, lung, ovary, nonregional node)

M1b: Metastases in more than one organ/site or the peritoneum

8.8.7.3 Tumor histology type

8.8.7.3.1 Histological type

Tubular adenocarcinoma, tub

Papillary adenocarcinoma, pap

Mucinous adenocarcinoma, muc

Signet ring cell carcinoma, sig

8.8.7.3.2 Histological grading

GX: rating cannot be evaluated

G1: highly differentiated

G2: differentiation

G3: poorly differentiated

G4: undifferentiated

8.8.7.4 Evaluation of tumor radical cure

8.8.7.4.1 Pathological assessment of margin

(1) Near cutting edge (PM)

PM(-): No infiltration of cancer was found near the margin

PM(+): cancer infiltration found near the margin

PM(x): Incomplete invasive marginal cancer infiltration

(2) Far cutting edge (DM)

DM(-): no cancer infiltration was found in the distal margin

DM(+): cancer infiltration found in distant margin

DM(x): Unclear marginal cancer infiltration is unknown

8.8.7.4.2 Record of radical cure

Postoperative tumor residuals were represented with R: R0 means radical resection and R1 and R2 mean non-radical resection.

RX: can't evaluate;

R0: no cancer residue;

R1: residual cancer microscopically (positive margin, positive peritoneal cytology);

R2: visible cancer residue.

8.8.7.5 Standardized evaluation of circumferential resection margin (CRM)

8.8.7.5.1 Assessment and grading of gross specimens around the circumference

The surgeon grades the quality of the TME procedure based on the circumferential margin of the gross specimen, usually in three levels:

- **Grade 3:** for the high-quality TME postoperative specimens, the mesorectal membrane is intact, the surface of the visceral fascia is not more than 5 mm, and the muscle wall of the intestinal wall is not seen.
- **Grade 2:** good TME postoperative specimen, that is, the rectal mesentery is intact, and there is a defect of more than 5 mm on the surface of the

visceral fascia. No intestinal muscle layer and sub-membranous incision margin is sufficient.

- **Grade 1:** for the gross specimen after poor TME, the intima of the mesorectum, the surface of the visceral fascia with more than 5 mm defect and the muscular layer of the intestinal wall.

8.8.7.5.2 Method for obtaining the circumferential resection margin

The specimen is cut along the opposite side of the tumor (for example, the tumor should be cut from the anterior wall), the intestinal wall and the mesentery are flattened, the mesangium is fixed upward on the foam plate, and the tumor is started from the nearest point, and the longitudinal axis of the parallel specimen is continuously cut. The thickness of 1 mm strip mesangial tissue is shown in the figure below.

9. ENDPOINTS AND DEFINITION

9.1 The research endpoints of this project are as follows:

Primary endpoints:

- Incidence of urinary dysfunction 2 weeks after surgery
- Incidence of sexual dysfunction 12 months after surgery

Secondary endpoint：

- 5-year overall survival rate
- Positive rate of circumferential resection margin
- Morbidity and mortality rates
- 1, 3-year overall survival rate
- 1, 3, 5-year disease free survival rate
- 1, 3, 5-year recurrence pattern
- Postoperative recovery course

9.2 Definition of the study endpoint for this project

9.2.1 Urinary dysfunction rate:

Two weeks after surgery, urinary dysfunction was identified as bladder residual urine volume >100 ml by ultrasound determination. The proportion of patients with urinary dysfunction in all patients is the rate of urinary dysfunction.

9.2.2 Sexual dysfunction:

At 12 months after operation, the patient was considered to suffer from moderate-severe erectile dysfunction when IIEF-5 score ≤11 points, or ejaculation dysfunction when the ejaculation function rating was at grade II or III.

9.2.3 3-year overall survival rate

Survival time goes from the starting point, which is the day of surgery, to either the time of death (independent of cause of death) or in case of survival, till the final date of survival confirmation. The 3-year survival rate refers to the proportion of patients that have survived for more than 3 years after various comprehensive treatments.

9.2.4 Incidence of surgical complications

The percentage of complications (listed in 8.4.5 above) occurred as a percentage of the total number of patients in each group, and was defined as the incidence of surgical complications. The severity of surgical complications was assessed according to the Clavien-Dindo classification I-V grading system.

Clavien-Dindo Classification:

Grade 1： any deviation from the normal postoperative course without the need for pharmacologic treatment or surgical, endoscopic, and radiologic interventions. Allowed therapeutic regimens are drugs as antiemetics, antipyretics, analgesics, diuretics, electrolytes and physiotherapy. This grade also includes wound infections opened at the bedside.

Grade 2： requiring pharmacologic treatment with drugs other than those allowed for grade I complications. Blood transfusions and total parenteral nutrition are also included.

Grade 3： requiring surgical, endoscopic, or radiologic intervention. 3a: intervention not under general anesthesia; 3b: intervention under general anesthesia.

Grade 4: life-threatening complication (including CNS complications) requiring IC/ICU management. 4a: single organ dysfunction (including dialysis); 4b: multiple organ dysfunction.

Grade 5： death as a result of complications.

(Abbreviations: CNS, central nervous system; IC, intermediate care; ICU, intensive care unit.)

9.2.5 Operative mortality

The percentage of deaths within 30 days (including day 30) after surgery as a percentage of the total number of patients in each group was defined as operative mortality.

9.2.6 Disease free survival (DFS)

With the study termination date as the cross-section, the number of cases without tumor recurrence as a percentage of the total number of cases per group was defined as the tumor-free survival rate.

9.2.7 Local recurrence rate and type of recurrence

The local recurrence rate is the percentage of tumor recurrence in the local area of the original surgery. The type of recurrence includes the intestinal wall, the surrounding pelvic wall, and the recurrence of the posterior wall of the seminal vesicle of the prostate.

9.2.8 Early postoperative recovery process indicators

The time to start ventilation and restoring fluid diet after surgery refers to the earliest time of venting and restoring fluid diet after the first postoperative operation, recorded in hours.

9.2.9 Transfer to open surgery

After the start of laparoscopic surgery, when laparoscopic surgery cannot be performed for any reason, the surgical approach is switched to open surgery for laparotomy. The percentage of cases in which the number of conversion cases in each group accounted for the total number of cases was the rate of conversion to open surgery.

9.2.10 Positive rate of circumferential margin

The pathological results showed that the number of cases with tumor infiltration within 1 mm of the tumor circumferential margin accounted for the percentage of the total number of patients in the group.

10. STATISTICAL ANALYSIS

The data were collected, and statistical analysis was performed using SPSS16.0 statistical software. In addition to the primary efficacy index, the difference between the incidence of urinary dysfunction and the incidence of sexual dysfunction was tested with a one-sided test, and other analyses were performed with a two-sided test. The statistical test level was set at 0.05, and the interval estimation of parameters was done using 95% confidence interval. Baseline data and validity analysis were analyzed using modified intention-to-treat (MITT) analysis. The main efficacy indicators were simultaneously analyzed by Per-Protocol Set (PPS), but were mainly based on the conclusions of the MITT analysis. The safety evaluation was analyzed by safety set (SS). Missing data are not filled. Quantitative data was analyzed using t-test, ANOVA, ANCOVA and non-parametric test. Qualitative data were analyzed using Pearson chi-square test, CMH test, and logistic regression. Grade data were analyzed using rank-based nonparametric methods; and survival data using Kaplan-Meier method (K-M) and Cox model. Sensitivity analysis was performed on extreme outlier data. Center effect analysis and subgroup analysis were performed when appropriate.

11. SUBJECT INFORMED CONSENT

The premise of the subject's participation in the study was his/her written informed consent.

The investigator must provide the subject with sufficient information before obtaining informed consent. In order to obtain informed consent, the investigator will provide a subject information page that is subject to applicable regulatory requirements. While providing written information, the investigator will verbally inform the subject of all relevant information about the study. In the process, the words used must enable non-experts to understand the information completely and easily, and to sign the informed consent form according to their own wishes and based on the subject's full understanding of the study. Informed consent must be signed and dated by the subject and the investigator. All subjects will be required to sign an informed consent form to prove their consent to participate in the study. The informed consent form with the name and date is kept at the research center where the researcher is located and must be kept in a safe place for inspection at any time. Subjects should be provided with a copy of their signed consent form, named and dated before they participate in the study. At any time during the study if there is important new information that is relevant to the subject's consent, the researcher will revise the information page and any other written information provided to the subject and submit it to the IEC/IRB for review. Revised information for consent will be provided to each subject participating in the study. The investigator will explain to the subject the modifications made to the previous version. Informed consent is attached.

12. ETHICS MATTERS

12.1 Researcher responsibilities

The investigator is responsible for the implementation of this study at its center. Researchers will ensure compliance with the *Helsinki Declaration* in accordance with research protocols. The research is carried out in accordance with domestic and international ethical guidelines and applicable regulatory requirements. It is important to note that the investigator must ensure that only subjects with informed consent are eligible for inclusion in the study.

12.2 Subject privacy

Immediately after obtaining informed consent, each enrolled subject is assigned an Allocation Number. This number will represent the subject throughout the study and in the clinical research database created for the study. Subject data collected during the study will be stored under this number.

Throughout the study, various security management measures will be taken to minimize the risk of data leakage during the use of personal information. Including: (1) after authorization, only the researcher can link the subject's research data to the person through the identification form held by the research center; (2) in the original data review conducted by the research monitor at the site, and in the relevant supervision department inspections,

the person conducting the above activities may review the original medical materials of the subjects, which will be kept strictly confidential.

The collection, transmission, processing and storage of subject data will comply with data protection and privacy regulations. The subject will be informed accordingly and the subject will be required to provide their consent to the above data processing procedures in accordance with national regulations.

12.3 Institutional ethics review committee

The study protocol and related documents (informed consent, subject information pages, CRF, and other documents that may be needed) will be submitted to the responsible independent ethics committee (IEC) / institutional review board (IRB) before the research center begins its research, to obtain their approval / disapproval. The IEC/IRB approval / disapproval file will be archived in the researcher's research center folder.

Research may not be initiated at the center until the investigator has obtained written support for the approval/disapproval of the relevant IEC/IRB. Written proof of the date of the approval meeting and written evidence of the members and voting members present at the meeting will be requested by IEC/IRB. IEC/IRB shall provide written evidence of its approval / disapproval, which shall document the study reviewed, the version of the protocol, and the version of the informed consent form. A copy of the minutes of the meeting

should also be obtained, if possible. In the event of major changes to the study, amendments to the study protocol will be submitted to the relevant IEC / IRB prior to implementation. Relevant safety information will be submitted to IEC / IRB in accordance with national regulations and requirements during the course of the study.

13. DESCRIPTION OF OTHER MATTERS

- **Preoperative neoadjuvant chemotherapy**: patients who underwent preoperative comprehensive evaluation and who were required to undergo preoperative neoadjuvant chemotherapy were also included in the study. Neoadjuvant therapy was used as a matching factor in randomization and stratified analysis was performed in the later outcome treatment. In view of the possible neurological damage caused by radiotherapy, neoadjuvant radiotherapy patients cannot be included in the study.
- **Postoperative adjuvant chemotherapy:** according to the postoperative staging of patients, adjuvant therapy should be used for high-risk stage II and III patients. XELOX and mFOLFOX6 are recommended for adjuvant chemotherapy. Units that are eligible for adjuvant radiotherapy can be taken.

14. DATA MANAGEMENT AND MONITORING SYSTEM

14.1 Case report form (CRF)

14.1.1 CRF submission

- Case screening: 7 days before surgery (time window 3 days);
- Enrollment: submit to the data center 1 day before surgery;
- Surgery: within 1 day after surgery;
- Postoperative-discharge: within 3 days after first discharge;
- Follow-up record: 7 days after each scheduled follow-up time.

14.1.2 Transfer of CRF

This study uses a paper version of the CRF form for the transmission of research data. The electronic version of CRF uses the Excel form that is uploaded via the Baidu cloud disk of each sub-center.

14.1.3 Revision of CRF

After the start of the study, if necessary data items or ambiguous items are found in the CRF, and the correction of the CRF will not cause the medical and economic burden or risk of the selected patients to increase, the research committee meets and discusses the correction of the CRF.

14.2 Process management and monitoring of the study

For the safety of the study and the implementation of the protocol, the data are correctly collected. The protocol is monitored monthly and the follow-up period is monitored every 2 months. The Clinical Research Management Committee (composed of the lead unit leader, the sub-center leader and the research secretary) conducts the monitoring of the research process by comparing the original data with the hospital visit. Meanwhile, the project was also monitored by the 5010 Project Management Department of Sun Yat-sen University every year.

Regular data reports completed by the data center are submitted to the research committee, research leader, and efficacy and safety evaluation committee. The data were discussed and analyzed in accordance with relevant regulations. The purpose of regular monitoring is to feedback problems and improve the scientific and ethical nature of research. It is not an attempt to expose research or hospital problems. The research committee, the research leader, and the research director of the research institute should work hard to improve and avoid the problems identified in the regular monitoring report.

14.2.1 Monitoring items

- Status of data collection completed: number of enrolled cases

(accumulated/different periods, all hospitals/different hospitals).

- Qualification: unqualified patients / potentially unqualified patients (different hospitals).
- Reasons for the end of the study/different treatment, suspension/end of the study plan (different hospitals).
- Background factors, pre-treatment reporting factors, post-treatment reporting factors.
- Serious adverse events (different hospitals).
- Adverse events / adverse reactions (different hospitals).
- Proportion of laparoscopic surgery (different hospitals).
- Open stomach transfer ratio (different hospitals).
- Agreement deviation (different hospitals).
- Urinary and sexual dysfunction, tumor-free survival / overall survival (all enrolled patients).
- Research progress and safety, other issues.

14.2.2 Acceptable range of adverse events (AE)

Adverse events should not occur, and if the proportion exceeds 3%, it is considered unacceptable. If you suspect that a death in this clinical trial, or the death and life-threatening complications caused by surgery, have a causal relationship with the surgery, report the adverse events of each patient to the

Committee on Efficacy and Safety (composed of research leader, heads of each branch, and research assistants). If treatment-related deaths or judgments have a causal relationship with surgery in 15 cases it is clear that the proportion of the final adverse events will exceed 3%, must be immediately discontinued whether it can continue the study, pending review by the Committee on Efficacy and Safety.

14.2.3 Deviation / violation of research protocols

Surgical resection, clinical examination or toxicity, efficacy evaluation, etc., which were not performed according to the research protocol, were deviations from the study protocol. In the case of monitoring, the data center and the research committee are ahead of time (in special cases, after the study is allowed to start), and the deviation of the acceptable range agreed in each study is formulated, and the monitoring report is included in the form of "cases with deviation possibility". After discussion by the research committee, it is divided into any of the following:

- **Violation:** a deviation that is clinically inappropriate and that meets at least one of the following items identified as "violation". They are listed below: the endpoint evaluation that affects the study, because of the doctor/hospital, deliberate or systematic, dangerous or deviating. In principle, the content of each violation should be recorded when the paper is published.

- **Acceptable deviation:** Deviation from the acceptable range set by the research representative/research committee and data center for each project before the study begins or after the study begins. Deviations within the acceptable range set in advance do not need to be recorded in the monitoring report.
- **Deviation:** a situation that does not meet the above two points is a deviation; when a specific deviation occurs multiple times, it is recorded as much as possible when the paper is published. When the monitoring report is discussed, it is classified as any one of the following: deviation from undesired results: should be reduced; deviation (inevitable): not to be actively reduced; deviation (clinically appropriate): positively affirming the judgment of the competent physician/hospital.

15. In order to protect the rights and interests of all parties involved in the research, this project has clearly stated and stipulated relevant intellectual property rights.

- Follow the established publication timeline principle in the research proposal when publishing the research results in the paper.
- In the case where there is no clear study group established policy when publishing the paper, follow the principle: the main statistical analysis, the

final statistical analysis, the main and final public papers are written, and the English journals are submitted. The main statistical analysis and final statistical analysis methods used cannot be published without the approval of the efficacy and safety evaluation committee, as specified in the protocol. However, the research representative or the research committee, which does not include the final statistical analysis results of this study, publishes the academic paper (outline) for the purpose of introducing the research, and can obtain the consent of the person in charge of the data center.

- In principle, the first author of the published paper is the head of research committee, followed by the research representative, the statistical person in charge of the data center (one person in charge of the statistical analysis for publication purposes). The rest follow the submission rules. According to the order of the number of selected registration samples, the people responsible for the research in the participating hospitals are listed as co-authors. All co-authors must review the content of the paper and agree to publish it before submitting it. The research representative has the right to exclude the researcher from being a co-author if he or she has no objection to the published content.
- For the overall data collected in this study, if the person in charge of the research unit needs secondary analysis or other research purposes for analysis, the research committee's consent is required. The person in

charge of the research unit needs to use the data of this research group, and also indicate the source of the data and inform the research committee.

- The publication of hypothesis-related research results for the main research purposes is in principle written by the research leader. The publication of the hypothesis-related research results or the secondary analysis results of the control data for the purpose of the secondary research may be negotiated by the person in charge of the research unit in the research organization, but the permission of the research leader is required.
- For single-center data of the participating units, the person in charge of the research unit has the right to keep the data of their center, but following the principle of privacy protection. For the results, forms and contents of the published single-center data, the responsibility of the person in charge in the publishing center shall be borne by the research center. The research representative/leader does not have any responsibility. When using single-center data, the data center should be informed and approve the data for accuracy. However, single-center data for statistical analysis should be indicated from this study to avoid repeated inclusion in system analysis.
- Both the Research Committee and the Data Center agree that publishers outside the Research Committee cannot obtain the overall data and

statistical analysis results of the study directly from the data center.

16. PRE-ASSESSMENT AND RISK CONTROL PLAN FOR PROJECT RISK BENEFITS

16.1 Relevant provisions for adverse events

16.1.1 Expected AE

(1) Postoperative complications of surgical-related adverse events: including intra-abdominal hemorrhage, gastrointestinal or anastomotic bleeding, anastomotic leakage, internal hemorrhoid formation, anastomotic stenosis, pelvic and abdominal infection, digestive tract infection, postoperative pneumonia, pleural effusion, pulmonary dysfunction, myocardial infarction, cardiac dysfunction, renal dysfunction, liver dysfunction, deep venous thrombosis of the lower extremities, cerebrovascular accident, coagulation or hematopoietic dysfunction.

(2) Adverse events caused by deterioration of primary disease

The relevant adverse events caused by various forms of exacerbation of the primary disease are recorded in detail according to CTCAE v3.0, including:

- Adverse events caused by deterioration of primary lesions and peritoneal disseminated lesions: digestive tract (loss of appetite, constipation, dehydration, fullness of the abdomen, heartburn, nausea, digestive tract

obstruction, digestive tract perforation, digestive tract stenosis, gastrointestinal bleeding).

- Adverse events caused by worsening liver metastasis: abnormal metabolic/clinical examination values (AST, ALT, bilirubin, alkaline phosphatase).
- Adverse events caused by worsening lung metastasis: lung/upper respiratory tract (atelectasis, difficulty breathing, hypoxemia, airway occlusion - [bronchial]).
- Other adverse events caused by the deterioration of metastatic lesions: pain: pain - [metastasis] (hypercalcemia).
- Adverse reactions caused by deterioration of general condition: fatigue, weight loss, cachexia, and other systemic adverse reactions.

16.1.2 Evaluation of adverse events

(1) Evaluation of adverse events/adverse reactions, comprehensive reference [Accordion Severity Grading System]

(2) When classifying adverse events, classify them according to the definitions closest to Grade0~4. In the case of treatment-related death, the adverse events of death were classified into Grade 5 in the original CTCAE.

(3) For the toxicity items specified in [Surgical-related adverse events], the date of discovery of Grade and Grade is recorded in the relevant recording

paper (treatment process report). For other toxicities, the toxicity items and the date of discovery of Grade and Grade were recorded in the free entry column of the treatment record paper only when Grade 3 or higher was observed. The Grade recorded in the recording paper must also be recorded in the case.

(4) The so-called "Adverse Event," "all adverse signs that are not expected to appear during treatment (abnormality of clinical examination values are included), symptoms, diseases, regardless of treatment or deal with whether or not there is a causal relationship, that is, to judge whether there is a causal relationship and a judgment of no causal relationship."

(5) Even if it is "caused by the primary disease (cancer)", it is not caused by the treatment of this study (protocol treatment) itself but by supportive therapy or combination therapy, which are all "adverse events".

(6) As a collection strategy for adverse event data, this study follows the following principles:

- Adverse events within 30 days of the last treatment day (protocol treatment) of this study protocol.
- The adverse events of the study protocol (therapy treatment) after 31 days from the last treatment day, only collect data that is causally related to protocol treatment.

16.1.3 Report of adverse events

When “severe adverse events (SAE)” or “unexpected adverse events” occur, the people responsible for the research in each participating unit should report to the Research Committee / PI (Wei Hongbo). The report style was sent to the participating units by the research committee prior to the start of the study.

Report to the provincial (city) health department of each research center based on relevant laws and regulations, report to the general manager of each medical institution based on serious adverse events related to the ethical guidelines for clinical research, and follow the relevant regulations of each medical institution to complete the corresponding reporting procedures. The research directors of each participating unit have the obligation and responsibility to urgently treat patients with any degree of adverse events to ensure patient safety.

(1) Obligation to report adverse events

1) Adverse events with urgent reporting obligations

- All deaths within 30 days of treatment or the last treatment day, regardless of whether or not there is a causal relationship with the treatment in this study. If the treatment is discontinued, even if the latter treatment has begun, as long as it is within 30 days of the last treatment

day, it is also an urgent report. “30 days” means the last day of treatment is the 0th day, starting from the next day (30 days of the number).

- Unexpected Grade 4 non-hematologic toxicity (adverse events other than blood/bone marrow grouping in CTCAE v3.0). A causal relationship with treatment (definite, probable, possible) is also an urgent report.

2) Adverse events with regular reporting obligations

- 31 days after the last treatment day, deaths that are causally related to treatment cannot be denied, including suspected treatment-related deaths. Significant primary disease deaths are not included.
- Expected non-hematologic toxicity of Grade 4 (adverse events other than blood/bone marrow grouping in CTCAE v3.0).
- Unexpected adverse events of Grade 3.
- Other major medical events: what the research team considers to be important and has the potential to cause permanent damage or adverse events that have a significant impact on the next generation. (except for MDS myelodysplastic syndrome, secondary cancer).

The latter three items, which are determined to have a causal relationship with the treatment of the study protocol (definite, probable, possible), belong to the regular report object.

(2) Reporting procedure

1) Emergency report

When an emergency report of an adverse event occurs, the competent doctor promptly reports to the researcher of the research institute. When it is not possible to get in touch with the person responsible for the hospital research, the hospital's contact person or the competent doctor must act as the person responsible for the hospital research.

- First report: within 72 hours of the adverse event, the hospital research person should complete the “AE/AR/ADR emergency first report” and send it to the research committee FAX.
- Second report: the researcher of each research institute completes the “AE/AR/ADR report”, which has a more detailed case information report (A4 format), and faxes two reports to the research within 15 days after the adverse event occurred. If an autopsy is performed, a report of the autopsy results is required.

2) Regular report

The hospital research person completes the “AE/AR/ADR report” and faxes it to the research committee within 15 days after the adverse event occurred.

16.2 Responsibility and obligations of the research leader / research committee

16.2.1 Judging the need for research suspension and emergency notification to the hospital

After receiving the report of the researcher responsible for the research institute, the research committee will reply to the researcher of the unit for confirmation and negotiation, jointly determine the urgency, importance, and degree of impact of the reported incident, and temporarily suspend the study if necessary. If necessary, they will contact all participating hospitals and emergency notifications. When contacting the data center or the research institute, you can use the telephone number according to the urgency, or you can contact the fax as soon as possible after the initial contact.

16.2.2 Report to the Committee on the Evaluation of Efficacy and Safety

The research committee deliberates and then informs the researcher in charge of the research unit, and clarifies that the adverse event in the emergency report or the routine report is consistent with “obligation to report adverse events”. The efficacy and safety evaluation committee report is written within 3 days after the adverse event occurs. The formal report also requests review of the reasons for the analysis of the adverse events and the appropriateness of the treatment.

At this time, the “AE/AR/ADR Emergency First Report” and “AE/AR/ADR Report” submitted by the Research Institute should include the discussion results and countermeasures of the research committee/research responsibility (including research continued/ Abort judgment). For deaths within 30 days, treatment-related deaths after 31st, and the expected non-hematotoxicity of Grade 4, not only the course of each patient, but also the frequency of occurrence is expected. When the frequency of occurrence exceeds the expected range, it must be recorded in the “II. Classification of Adverse Events - Others” in the “AE/AR/ADR Report”.

16.2.3 Notice to the research institute

After submitting the report to the efficacy and safety evaluation committee, the research committee/responsible person shall notify all hospitals participating in the study of the review and recommendations of the efficacy and safety evaluation committee in writing.

If the report is not submitted to the efficacy and safety evaluation committee, the research committee/responsible person shall notify the researcher responsible for the report to the research committee/research person in writing.

16.2.4 Regularly monitored AE discussion

At the time of regular monitoring, the research committee/responsible person should carefully discuss and study the adverse events in the monitoring report submitted by the data center, and confirm that there are no false reports in each participating hospital. The presence or absence of false negatives is clearly recorded in the discussion of the [Regular Monitoring Report] by the Research Committee.

16.2.5 Review of the Committee on Efficacy and Safety Evaluation

The Committee on Efficacy and Safety evaluates and discusses the content of the report in accordance with the procedures documented in the Guidelines for the Management of Clinical Safety Information, and suggests in writing to the person responsible for the study whether it is possible to continue to be included in the study or whether the study protocol needs to be revised.
