# Supplementary material for: Preservation versus resection of Denonvilliers’ fascia in total mesorectal excision for male rectal cancer: follow-up analysis of the randomized PUF-01 trial
Source: Nat Commun. 2023 Oct 20;14:6667. doi: 10.1038/s41467-023-42367-3 (PMC10589235; doi:10.1038/s41467-023-42367-3)
Supplement: Supplementary file 2 — Reporting Summary [file 41467_2023_42367_MOESM2_ESM.pdf]

nature portfolio

Corresponding author(s): Hongbo Wei

Last updated by author(s): Oct 4, 2023

Reporting Summary

Nature Portfolio wishes to improve the reproducibility of the work that we publish. This form provides structure for consistency and transparency in reporting. For further information on Nature Portfolio policies, see our Editorial Policies and the Editorial Policy Checklist.

Statistics

For all statistical analyses, confirm that the following items are present in the figure legend, table legend, main text, or Methods section.

n/a	Confirmed	
[ ]	[x]	The exact sample size (*n*) for each experimental group/condition, given as a discrete number and unit of measurement
[x]	[ ]	A statement on whether measurements were taken from distinct samples or whether the same sample was measured repeatedly
[ ]	[x]	The statistical test(s) used AND whether they are one- or two-sided<br>*Only common tests should be described solely by name; describe more complex techniques in the Methods section.*
[x]	[ ]	A description of all covariates tested
[x]	[ ]	A description of any assumptions or corrections, such as tests of normality and adjustment for multiple comparisons
[ ]	[x]	A full description of the statistical parameters including central tendency (e.g. means) or other basic estimates (e.g. regression coefficient) AND variation (e.g. standard deviation) or associated estimates of uncertainty (e.g. confidence intervals)
[ ]	[x]	For null hypothesis testing, the test statistic (e.g. *F*, *t*, *r*) with confidence intervals, effect sizes, degrees of freedom and *P* value noted<br>*Give P values as exact values whenever suitable.*
[x]	[ ]	For Bayesian analysis, information on the choice of priors and Markov chain Monte Carlo settings
[x]	[ ]	For hierarchical and complex designs, identification of the appropriate level for tests and full reporting of outcomes
[ ]	[x]	Estimates of effect sizes (e.g. Cohen's *d*, Pearson's *r*), indicating how they were calculated

Our web collection on statistics for biologists contains articles on many of the points above.

Software and code

Policy information about availability of computer code

Data collection	Microsoft Excel (Microsoft Corporation, 2021)
Data analysis	Statistical analysis was performed using the SPSS 25.0 statistical software (IBM Corp. USA) and R, version 3.6.2 (R Group for Statistical Computing).

For manuscripts utilizing custom algorithms or software that are central to the research but not yet described in published literature, software must be made available to editors and reviewers. We strongly encourage code deposition in a community repository (e.g. GitHub). See the Nature Portfolio guidelines for submitting code & software for further information.

Data

Policy information about availability of data

All manuscripts must include a data availability statement. This statement should provide the following information, where applicable:

- Accession codes, unique identifiers, or web links for publicly available datasets
- A description of any restrictions on data availability
- For clinical datasets or third party data, please ensure that the statement adheres to our policy

De-identified and processed participant data will be shared beginning 3 months and ending 5 years following publication by requesting the corresponding author (Hongbo Wei, E-mail: weihb@mail.sysu.edu.cn) for academic purposes. The corresponding author will reply to the request within 2 months, subject to the approval of the ethics committees of the Third Affiliated Hospital, Sun Yat-Sen University. Source data underlying Figs. 2-3 are provided with this paper. The study protocol is available as a supplementary file (Supplementary Note).

Research involving human participants, their data, or biological material

Policy information about studies with human participants or human data. See also policy information about sex, gender (identity/presentation), and sexual orientation and race, ethnicity and racism.

Reporting on sex and gender	Our research only included male patients with rectal cancer. First because the structure of DVF is more complicated and multiple-morphologic for female, thus feasibility of DVF preservation for female is still unclear. In addition, the assessment method of sexual function for female is relatively insufficient. Thus, we did not enroll female rectal cancer patients in this study. Meanwhile, gender of participants was determined based on self-report.
Reporting on race, ethnicity, or other socially relevant groupings	This study is conducted in China and thus only Chinese patients were included.
Population characteristics	Male, 20 < age (years) < 71
Recruitment	When male rectal cancer patients were diagnosed in the 11 medical centers in this study, principal investigator (PI) or his/her team members would evaluate whether the patient met the inclusion criteria. If yes, they will talk with the patient, invite him to participate in this trial. After written informed consent was obtained, the patient would be included in this trail.
Ethics oversight	The protocol was approved by the Ethics Committee of the Third Affiliated Hospital, Sun Yat-Sen University.

Note that full information on the approval of the study protocol must also be provided in the manuscript.

Field-specific reporting

Please select the one below that is the best fit for your research. If you are not sure, read the appropriate sections before making your selection.

- [x] Life sciences
- [ ] Behavioural & social sciences
- [ ] Ecological, evolutionary & environmental sciences

For a reference copy of the document with all sections, see nature.com/documents/nr-reporting-summary-flat.pdf

Life sciences study design

All studies must disclose on these points even when the disclosure is negative.

Sample size	In this study, the incidences of urinary dysfunction 2 weeks postoperatively and sexual dysfunction 12 months postoperatively were the primary endpoints and dominant evaluation indicators. In our previous study, the incidence of urinary dysfunction and sexual dysfunction were 24.39% and 9.76%, respectively, for DVF-preserving procedure; the corresponding incidences for DVF-resecting procedures were 44.68% and 42.55%. According to the superiority study design, the sample size was determined using an alpha of 5% and 0.025 as the unilateral statistical significance level, setting the test efficiency to 90%. The final sample size takes the maximum 1 from the 2 indicators. At least 110 patients were required in each group. The sample size was calculated using the SAS 9.3 software.
Data exclusions	In this study, cases of APR or Non-R0 resection were excluded for per-protocol analysis of postoperative urogenital function. This is because that APR may have influence on postoperative urogenital function. Also, Non-R0 resection usually requires additional radiotherapy, which also have potential adverse effects on urogenital function. Nevertheless, these patients were still included in intention-to-treat analysis for oncological outcomes.
Replication	DVF began with a white thickened line at the lowest level of peritoneal reflection, and ended at the perineal body. Dissection below this marker line leads to entry posterior to DVF easily, and thus DVF could be entirely preserved. Even for patients with high BMI or narrow pelvic cavity, hanging the peritoneal reflection with a suture or performing traction of the rectum with tieback will help better exposure of both the pelvic cavity and the anterior wall of rectum, and thus make surgery easier to generalize. Thus, laparoscopic TME with DVF preservation is technically reproducible.<br>Both surgical videos and photographs of each case were mandatorily demanded to verify the reproducibility of this surgical approach. In this study, eleven Chinese colorectal surgeons from 11 institutions satisfying the following criteria were selected: (1) the surgeons had performed at least 50 L-TME procedures, (2) their surgical technique and radical resection skills were recognized by an Academic Committee. Thus, for surgeons who are well trained, this surgical procedure is feasible and reproducible.

Randomization	Stratified blocked randomization was used, stratification factor was center, and the block size was 4. The random allocation sequence was generated by a statistician who was independent of the research, using the SAS 9.3 software (SAS Institute, Cary, NC). Participants were randomly assigned using random envelopes in a 1:1 ratio to groups that underwent laparoscopic TME with DVF preservation (L-DVF-P, Exp-group) or DVF resection (L-DVF-R, Con-group), respectively.
Blinding	The surgeons were informed of grouping information preoperatively, while the participants and research assistants enrolling in patient follow-up and functional evaluation were blinded.

Reporting for specific materials, systems and methods

We require information from authors about some types of materials, experimental systems and methods used in many studies. Here, indicate whether each material, system or method listed is relevant to your study. If you are not sure if a list item applies to your research, read the appropriate section before selecting a response.

Materials & experimental systems

n/a	Involved in the study	
☒	☐	Antibodies
☒	☐	Eukaryotic cell lines
☒	☐	Palaeontology and archaeology
☒	☐	Animals and other organisms
☐	☒	Clinical data
☒	☐	Dual use research of concern
☒	☐	Plants

Methods

n/a	Involved in the study	
☒	☐	ChIP-seq
☒	☐	Flow cytometry
☒	☐	MRI-based neuroimaging

Clinical data

Policy information about clinical studies

All manuscripts should comply with the ICMJEguidelines for publication of clinical research and a completedCONSORT checklist must be included with all submissions.

Clinical trial registration	ClinicalTrials.gov Registration: NCT02435758.
Study protocol	Attached with file named :Supplementary Note"
Data collection	Patients were enrolled from August 26, 2015, through May 6, 2020. The last follow-up was on October 24th, 2022.<br>The patients' urinary function was evaluated by RUV (mL, by ultrasonography), maximum flow rate (MFR, mL/s, by urodynamics), and International Prostate Symptom Score (IPSS). Erectile function and ejaculation function were evaluated by IIEF-5 and ejaculation function grading (Grade I: normal ejaculation; Grade II: retrograde ejaculation; Grade III: anejaculation), respectively. These data were collected from the medical records.<br>The oncological data included the 3-year overall survival (OS) and disease-free survival (DFS), and recurrence rate. Postoperative follow-up was performed every 3 months within 2 years, and every 6 months 3–5 years postoperatively. Comprehensive hematology, chest and abdomen spiral CT, and colonoscopy were used to evaluate the patient's postoperative survival status. OS was calculated from the day of randomization until the day of death (event) or the day of the last follow-up examination (censored), while DFS was calculated from the day of randomization until the day of recurrence or death (event) or the day of the last follow-up examination (censored). Data were censored for patients with no evidence of diseases at the last follow-up examination or for patients who died from other diseases or reasons without evidence of recurrence. These data were collected through follow-up by outpatient clinic, telephone, or E-mail.
Outcomes	The primary endpoints were incidences of urinary dysfunction 2 weeks postoperatively and sexual dysfunction 12 months postoperatively. The patients' urinary function was evaluated by RUV (mL, by ultrasonography), maximum flow rate (MFR, mL/s, by urodynamics), and International Prostate Symptom Score (IPSS). Erectile function and ejaculation function were evaluated by IIEF-5 and ejaculation function grading (Grade I: normal ejaculation; Grade II: retrograde ejaculation; Grade III: anejaculation), respectively.<br>The secondary endpoints were 3-year overall survival (OS), disease-free survival (DFS), and recurrence rate. Postoperative follow-up was performed every 3 months within 2 years, and every 6 months 3–5 years postoperatively. Comprehensive hematology, chest and abdomen spiral CT, and colonoscopy were used to evaluate the patient's postoperative survival status. OS was calculated from the day of randomization until the day of death (event) or the day of the last follow-up examination (censored), while DFS were calculated from the day of randomization until the day of recurrence or death(event) or the day of the last follow-up examination (censored). Data were censored for patients with no evidence of diseases at the last follow-up examination or for patients who died from other diseases or reasons without evidence of recurrence. The last follow-up was on October 24th, 2022.
